# Effect of Ammonium:Nitrate Application Ratios on Growth and Nitrogen Metabolism of Tea Plants ( *Camellia sinensis* L.)

**DOI:** 10.1002/pld3.70084

**Published:** 2025-06-14

**Authors:** Takuo Enomoto, Natsuki Tone, Takaya Ishii, Hisako Hirono, Ayako Oi, Yuhei Hirono, Takashi Ikka, Hiroto Yamashita

**Affiliations:** ^1^ Division of Tea Research Institute of Fruit Tree and Tea Science, National Agriculture and Food Research Organization (NARO) Shimada Shizuoka Japan; ^2^ United Graduate School of Agricultural Science Gifu University Gifu Japan; ^3^ Research Institute of Green Science and Technology Shizuoka University Shizuoka Japan; ^4^ Faculty of Agriculture Shizuoka University Shizuoka Japan; ^5^ Institute of Tea Science Shizuoka University Shizuoka Japan

**Keywords:** amino acids, ammonium, nitrate, tea, transcriptome

## Abstract

Tea plants ( *Camellia sinensis* L.) use ammonium and nitrate as the main sources of nitrogen (N), but they respond differently to these two compounds. In this study, we investigated the effect of the ammonium:nitrate ratio on tea plant growth as well as N uptake and metabolism. A kinetics analysis showed that both ammonium and nitrate were absorbed, with no major differences within the concentration range 0.71–2.86 mM. Additionally, growth peaked when the ammonium:nitrate ratio was 25:75. The concentrations of several free amino acids, including theanine, in new leaves and roots increased as the proportion of ammonium increased. Glutamine concentrations in new leaves and roots were highest at ammonium:nitrate ratio of 25:75. Moreover, the transcription of key genes involved in theanine and glutamine biosynthesis was differentially affected by changes in N ratios, which explained the differences in metabolic changes. The glutamine:theanine ratio was higher at an ammonium:nitrate ratio of 25:75 than at 100:0 and 75:25, suggesting that the ammonium:nitrate ratio may affect the ratio of glutamine synthesis activity to theanine synthesis activity. We examined N metabolism regulatory genes and identified candidate genes, including *SENSITIVE TO PROTON RHIZOTOXICITY 3.1* and *NITRATE‐INDUCIBLE GARP‐TYPE TRANSCRIPTIONAL REPRESSOR 1.2*, in tea plants. These transcription factor genes are involved in the regulation of nitrate absorption and metabolism. Identifying genes that regulate N metabolism is essential for improving N use efficiency. The study findings will be useful for optimizing N fertilization management practices to control tea growth and quality.

## 1 Introduction

Crop productivity is highly influenced by nitrogen (N) fertilization. Ammonium and nitrate are the main forms of N available to plants in soil. These two N sources differentially affect plant physiology and development (Min et al. 1999). Comparative studies involving various plants revealed preferences for ammonium or nitrate are usually related to the physiological adaptation of the plant to the natural ecosystem (Gerendás et al. 1997). Ammonium is toxic to most plant species (Britto and Kronzucker 2002) but some species that preferentially grow in wetland conditions (e.g., paddy soils and acidic soils) can adapt to high ammonium conditions (Nakamura and Nakamura 2012; Britto and Kronzucker 2013; Van den Berg et al. 2005).

Tea plants ( *C. sinensis* L.), which prefer ammonium over nitrate, can absorb more ammonium than nitrate (Ishigaki 1974; Ruan et al. 2016). Furthermore, total N and free amino acid (FAA) contents are higher in plants treated with ammonium than in plants treated with nitrate (Ishigaki 1974; Yang et al. 2013; Y. Wang, Wang, et al. 2021). However, tea plants require nitrate, with better growth reported for tea plants fertilized with both ammonium and nitrate than for tea plants treated with either N source alone (Ishigaki 1978). There has been relatively little research that simultaneously evaluated tea productivity and quality under cultivation conditions that differ in terms of ammonium and nitrate concentrations, and their relationship remains unclear.

Depending on whether the N source is ammonium or nitrate, the expression of genes involved in amino acid synthesis is controlled, thereby affecting the composition of FAAs, which are important tea quality determinants (Y. Wang, Wang, et al. 2021). Theanine (γ‐glutamylethylamide), which is the most abundant FAA in tea plants, was first identified in tea leaves (Sakato 1950). Tracer experiments revealed that theanine is synthesized in the roots (Deng and Ashihara 2015). Theanine is then translocated to aerial plant parts and contributes to the N supply (Dong et al. 2020). In tea plants, theanine biosynthesis is mediated by the enzyme encoded by *THEANINE SYNTHETASE I* (*CsTS I*), with the *CsTS I* expression pattern closely correlated with the theanine concentration (She et al. 2022). Theanine concentrations increase with increases in the application of ammonium (Ishigaki 1978). This indicates that ammonium fertilization may lead to increased *CsTS I* transcription. GLUTAMINE SYNTHETASE (GS), a key enzyme for N assimilation, is essential for glutamine synthesis, with the amide moiety of glutamine serving as an amino donor for the synthesis of other amino acids, proteins, and N‐containing biological molecules required by all living organisms (Chellamuthu et al. 2014). *CsGS* and *GLUTAMATE SYNTHASE* (*CsGOGAT*) expression levels in the new roots of tea plants tend to be higher after a treatment with ammonium than after a treatment with nitrate (Y. Wang, Wang, et al. 2021). However, because these previous reports were for short‐term treatments (a few hours or days), the effects of long‐term treatments (over several months) will need to be evaluated.

Several studies on Arabidopsis and rice have clarified the mechanisms regulating gene expression and activity in nitrate and ammonium metabolic pathways. In Arabidopsis, *NITRATE TRANSPORTER* (*AtNRT*) *1.1*, *AtNRT2.1*, *AtNRT2.2*, *NITRATE REDUCTASE* (*AtNR*) *1*, *AtNR2*, and *NITRITE REDUCTASE 1* (*AtNIR1*) expression is activated by the transcription factors NIN LIKE PROTEIN (AtNLP), TGACG MOTIF‐BINDING FACTOR (AtTGA) 1, AtTGA4, BASIC LEUCINE‐ZIPPER 1 (AtbZIP1), TCP‐DOMAIN FAMILY PROTEIN 20 (AtTCP20), and SENSITIVE TO PROTON RHIZOTOXICITY 1 (AtSTOP1) (Konishi and Yanagisawa 2013; Alvarez et al. 2014; Para et al. 2014; Guan et al. 2014; Tokizawa et al. 2023). Transcription factors that suppress expression include LOB DOMAIN‐CONTAINING PROTEIN (AtLBD) 37, 38, 39, and NITRATE‐INDUCIBLE GARP‐TYPE TRANSCRIPTIONAL REPRESSOR 1 (AtNIGT1) (Rubin et al. 2009; Maeda et al. 2018). In terms of ammonium absorption, in rice, SERINE/THREONINE/TYROSINE PROTEIN KINASE 1 (OsACTPK1) directly phosphorylates and inactivates AMMONIUM TRANSPORTER (OsAMT) 1;1 and OsAMT1;2 (Beier et al. 2018). The regulatory mechanisms involving these proteins are believed to be conserved in tea, which is a higher plant species. There are also several reports of N metabolism gene regulators in tea plants. CsLBD37, 38, and 39 have been identified in tea plants; the overexpression of *CsLBD37* in Arabidopsis suppresses *AtNR* and *AtNRT1.1* expression (Teng et al. 2022). A number of transcription factors that regulate N metabolism in tea plants have been reported. CsMYB73 is a transcriptional repressor that regulates the expression of *CsGS1* and *CsGS2* (Wen et al. 2020). In the theanine synthesis pathway, CsMYB6 regulates the expression of *CsTS I* (Zhang et al. 2021b), while CsHHO3 (CsNIGT1.1) and MYB40 regulate the expression of an alanine decarboxylase gene (*CsAlaDC*) (Guo et al. 2022).

To characterize variations in N metabolism, additional longer‐term analyses involving different N forms are required, with potential implications for producing high‐quality tea. In this study, we used a hydroponic experimental system to analyze the effects of different ammonium:nitrate application ratios on tea plant growth and N metabolism. Furthermore, a transcriptome analysis was conducted to clarify the factors affecting growth and N metabolism. The study results will contribute to improving the balance between tea growth and quality.

## 2 Materials and Methods

### 2.1 Plant Growth

**FIGURE 1:**
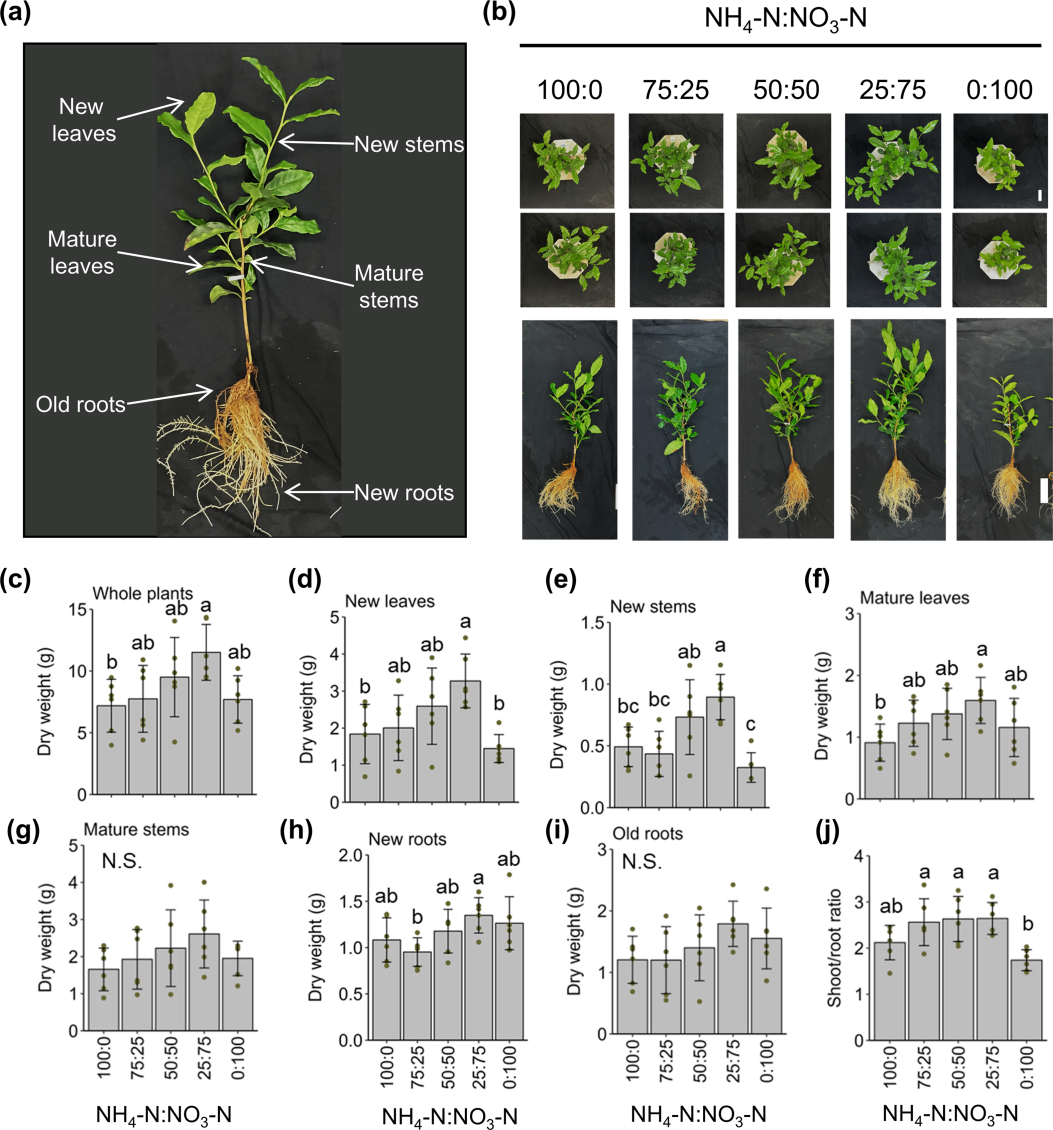
Effect of inorganic nitrogen (N) source ratios on tea plant growth. (a) Appearance of 1‐year‐old tea plants and their organs. (b) Appearance of tea plants grown in this study. (c–i) Dry weight of (c) whole plants, (d) new leaves, (e) new stems, (f) mature leaves, (g) mature stems, (h) new roots, and (i) old roots. (j) Shoot:root ratios (according to dry weight). Data are presented as the mean ± SD Dots represent replicates (*n* = 6). Different letters indicate significant differences as determined by Tukey's HSD test (*p* < 0.05). N.S., not significant (*p* ≥ 0.05). White bar = 10 cm.

Tea plants were cultured in a hydroponic system under ambient light in an unheated greenhouse (120 m^2^) at Shizuoka University (Shizuoka, Shizuoka, Japan), with an average temperature of 20 °C in spring (late March to late June) 2023. A slightly modified version of the culture method described by Konishi et al. (1985) was used. Briefly, 1‐year‐old rooted tea cuttings of ‘Yabukita’, a leading Japanese green tea cultivar, were transplanted to Wagner pots (size: 1/5000; diameter: 159 mm; depth: 190 mm) containing 3 L tap water adjusted to pH 4.2 and continuously aerated. Plants used for analyses of growth, metabolites, and transcription were grown and treated with the following inorganic N:NH_4_ and N:NO_3_‐N ratios: 100:0 (2.86 mM:0 mM), 75:25 (2.14 mM:0.71 mM), 50:50 (1.43 mM:1.43 mM), 25:75 (0.71 mM:2.14 mM), and 0:100 (0 mM:2.86 mM). Additional details regarding solution compositions are provided in Supplementary Tables 1–5. Treatment solutions were renewed once per week. After 4 months, tea plants were harvested (13 June 2023), washed with deionized water, wiped dry, and separated into the following parts: new leaves (leaves from the second tea shoot that grew after the first tea shoot was plucked on 18 April 2023), new stems, mature leaves (remaining non‐new shoots), mature stems, old roots (brown roots), and new roots (white roots) (Figure 1a). Fresh samples were weighed and then freeze‐dried. Additionally, leaves from two new shoots at the top of plants and 100 mg root tips (20 mm) were frozen and ground for the subsequent analysis of transcription. Freeze‐dried samples were ground to a fine powder before measuring the total N, ammonium, nitrate, and FAA contents. Plants used for the kinetics analysis were grown for 4 months as described by Konishi et al. (1985) (Supplementary Table 6).

### 2.2 Influx Kinetics Analysis

^15^NH_4_^+^ and ^15^NO_3_^−^ influx kinetics were measured as a function of external ammonium and nitrate concentrations. A flow chart for the influx kinetics experiment is presented in Supporting Figure 1. Four replicates of tea plants per treatment were placed in a 1 mM CaSO_4_ rinse solution and preincubated at 25 °C in a growth chamber for 10 min under light. Each treatment was completed using four plants grown in separate pots. They were transferred to nutrient solutions containing either (^15^NH_4_)_2_SO_4_ (^15^N abundance = 99.9%) or K^15^NO_3_ (^15^N abundance = 99.9%) as the only N source and incubated at 25 °C in a growth chamber for 10 min. External ^15^NH_4_^+^ or ^15^NO_3_^−^ concentrations in this solution were 0.01, 0.02, 0.05, 0.1, 0.2, 0.3, 0.4, 0.5, 0.75, 1, 2, 5, and 10 mM. The concentration range with the largest changes in an earlier study by Yang et al. (2013) was used as a reference. After each treatment, plant roots were thoroughly washed using the rinse solution for 1 min. The roots were divided into new roots and old roots, with the new roots used for analyses. Specifically, new roots were stored at −20 °C and then freeze‐dried. They were subsequently homogenized to a fine powder for the analysis of the labeled N concentration.

Plant samples were enclosed in a tin cup and combusted using an elemental analyzer (FlashEA1112; Thermo Fisher Scientific, Waltham, Massachusetts, USA) interfaced with an isotope ratio mass spectrometer (Thermo Scientific Delta V Advantage; Thermo Fisher Scientific), which was used to analyze N isotope ratios.

Using substrates labeled with ^15^N, we analyzed uptake kinetics using different initial substrate concentrations. The influx into tea plant roots reportedly follows a classic biphasic pattern, with high‐affinity and low‐affinity transporters (Yang et al. 2013). We assumed only the high‐affinity transporter was active below a certain substrate concentration and the low‐affinity transporter was activated above a certain substrate concentration. The following two‐step Michaelis–Menten curves were plotted, corresponding to the uptake rate for the analyzed concentrations.

$$v=\left\{\begin{array}{c} \frac{V_{1}\left[S\right]}{K_{m1}+\left[S\right]}\;\left(\left[S\right]<T\right)\\ \frac{V_{1}T}{K_{m1}+T}+\frac{V_{2}\left(\left[S\right]-T\right)}{K_{m2}+\left(\left[S\right]-T\right)}\;\left(\left[S\right]\geq T\right) \end{array}\right.$$

*V*_*1*_ and *V*_*2*_ are the maximum reaction velocities of the high‐affinity and low‐affinity transporters, respectively, *K*_*m1*_ and *K*_*m2*_ are the respective Michaelis constants, *S* is the substrate concentration, and *T* is the concentration at which the low‐affinity transporter was activated. A nonlinear least squares analysis was performed using python 3.9.16 and curve fit from the SciPy 1.10.1 package.

### 2.3 RNA Extraction and RNA‐Seq Analysis

We extracted RNA from new leaves and new roots using separate RNA extraction kits because of the difference in extraction efficiency between these tissues. Specifically, total RNA was extracted from new leaves using a Plant RNA Extraction Mini kit (QIAGEN, Japan) and from new roots using an ISOSPIN Plant RNA kit with Assist Buffer (NIPPON GENE, Japan) according to the manufacturers' instructions. The extracted RNA was qualitatively analyzed by 1.5% agarose gel electrophoresis. In addition, the RNA concentration was determined using a nucleic acid concentration meter (Nanovue; GE HealthCare, Tokyo, Japan). High‐quality RNA was sent to Clockmics Inc. (Osaka, Japan) for library construction and sequencing. Reads were preprocessed using Fastp v0.23.4 (Chen et al. 2018) and the remaining reads were mapped to the tea reference genome (Kawahara et al. 2024), which was the Seimei genome v1.2 (https://agrigenome.dna.affrc.go.jp/tasuke/Tea_Seimei/) downloaded from TASUKE+, using STAR v2.7.10b (Dobin et al. 2013). Read counts were calculated using RSEM v1.3.3 (Li and Dewey 2011) and analyzed in the R environment using the edgeR package. Genes with a mean read count <10 per sample were filtered out to eliminate genes expressed at low levels. Read count data for the remaining genes were normalized using the TMM method and the function “calcNormFactors”. Data were log_2_ transformed using the function CPM (counts per million) to yield log_2_ CPM values.

A K‐means clustering analysis was completed using the “K‐means” function in the R package and the RNA‐seq dataset for new leaves. For the Gene Ontology (GO) enrichment analysis, the eggnog mapper was used to annotate the tea reference genome with GO terms (Cantalapiedra et al. 2021). Specifically, the GO enrichment analysis was performed using the “runTest” function in the R package “topGO” v2.40.0 and the following options: algorithm = “elim” and statistic = “fisher”. Enriched GO terms with a false discovery rate < 0.05 were considered significant. UpSet plots were prepared using the “UpSet” function in the R package “UpSetR” v1.4.0.

### 2.4 Total N and FAA Concentration Analysis

The total N content was analyzed as described by Hirono and Nonaka (2014), whereas the total N content was determined using an elemental analyzer (Flash EA 1112; Thermoquest, Milan, Italy). FAAs were analyzed as described by Yamashita et al. (2021). Specifically, dry ground plant tissue (10 mg) was mixed with 10 mg polyvinylpolypyrrolidone and 5 mL ultrapure water for a 60‐min extraction at room temperature with shaking (130 strokes per min). The extract was centrifuged (2000 × *g* for 15 min at 4 °C) and then the supernatant was passed through a 0.45‐μm cellulose acetate filter (ADVANTEC, Tokyo, Japan). The FAA concentration was determined using a high‐performance liquid chromatography system (Shimadzu, Tokyo, Japan).

### 2.5 Nitrate and Ammonium Concentration Analysis

Nitrate concentrations were analyzed as described by Yamashita et al. (2024). The aqueous extract was analyzed by ion chromatography. A pretreatment step for the ammonium concentration analysis was performed as described by Bräutigam et al. (2007), with some modifications. Dry ground plant tissue (10 mg) was mixed with 1 mL hydrochloric acid solution (100 mM) and then 500 μL chloroform was added. Samples were rotated for 15 min at room temperature, after which the phases were separated by centrifugation (12,000 × *g* for 10 min at 8 °C). The aqueous phase was transferred to a fresh tube containing 50 mg acid‐washed activated charcoal, thoroughly mixed, and centrifuged (20,000 × *g* for 5 min at 8 °C). Ammonium concentrations were analyzed using LabAssay Ammonia (Wako, Tokyo, Japan). To measure ammonium quantities, 80 μL supernatant obtained after the charcoal treatment was transferred to a 1.5 mL centrifuge tube and mixed with 320 μL protein removal reagent. After centrifuging the homogenate (5000 × *g* for 15 min at 4 °C), 300 μL supernatant was transferred to a 1.5 mL centrifuge tube. Color reagents A, B, and C were added and the homogenate was incubated at 37 °C for 20 min. Samples were left undisturbed at room temperature for 30 min before measuring the absorbance at 625 nm using a spectrophotometer. The ammonium concentration was calculated according to a standard curve.

### 2.6 Statistical Analysis

Statistical analyses were conducted using R v4.4.1 software. Tukey's HSD test was used for multiple comparisons of growth and metabolite phenotypes as well as gene expression. The threshold for significance was set at *p* < 0.05 (95% confidence interval).

Regression modeling of phenotypic data and RNA‐seq data for new leaves was completed as described by Yamashita et al. (2023). All data for each treatment were divided into training (70%) and test (30%) datasets using a stratified sampling approach. Non‐informative transcriptome data were eliminated using the Boruta feature selection algorithm (Kursa et al. 2010). More specifically, the following parameters of the “Boruta” function in the R package Boruta v8.00 were applied: getlmp = getlmpRfZ, maxRuns = 1000, and *p* = 0.01. This feature selection process was repeated 100 times to ensure the results were robust. For regression modeling, the informative transcriptome was used to generate 10–100 genes (at intervals of 10) in 100 replicates. Regression models were then constructed using the informative transcriptome and random forest algorithms (method = “rf”) in the R package “caret” v7.0.1. The hyperparameter “mtry” was optimized by a random search involving a repeated cross‐validation using the following parameters: number = 10, repeats = 3, and tuneLength = 10. To assess the accuracy of predictions, the coefficient of the determination (R^2^), root‐mean‐square error, and mean absolute error were calculated for the observed and predicted values. The black‐box model was interpreted according to variable importance and partial dependence plots. Variable importance was calculated using the “varlmp” function in the R package “caret”, whereas partial dependence plots were generated using the “partial” function in the R package “pdp” v0.8.2.

## 3 Results

### 3.1 Effects of Inorganic N Sources on Tea Plant Growth

We evaluated the growth of 1‐year‐old rooted tea cuttings grown under hydroponic conditions and treated with inorganic N sources at different ratios; the control ammonium:nitrate ratio (75:25) was set on the basis of the control used by Konishi et al. (1985) (Figure 1b). There were differences in the whole plant dry weight (DW), which increased when both ammonium and nitrate were included in the treatment (Figure 1c). Specifically, an ammonium:nitrate ratio of 25:75 resulted in the highest values. Growth differences were particularly evident for new leaves, new stems, and mature leaves, with the 25:75 treatment producing the highest values (Figure 1d, e, f). There were also significant differences in the growth of new roots among treatments, but the differences were not as large as the differences in the growth of new leaves, new stems, and mature leaves (Figure 1h). The effects of the various treatments on mature stems and old roots did not differ significantly (Figure 1g, i). Interestingly, the aerial parts: roots ratio tended to be higher for treatments that included both ammonium and nitrate than for treatments in which only ammonium or nitrate was applied (Figure 1j).

### 3.2 ^15^NH_4_^+^ and ^15^NO_3_^−^ Influx Kinetics of Tea Plants

**FIGURE 2:**
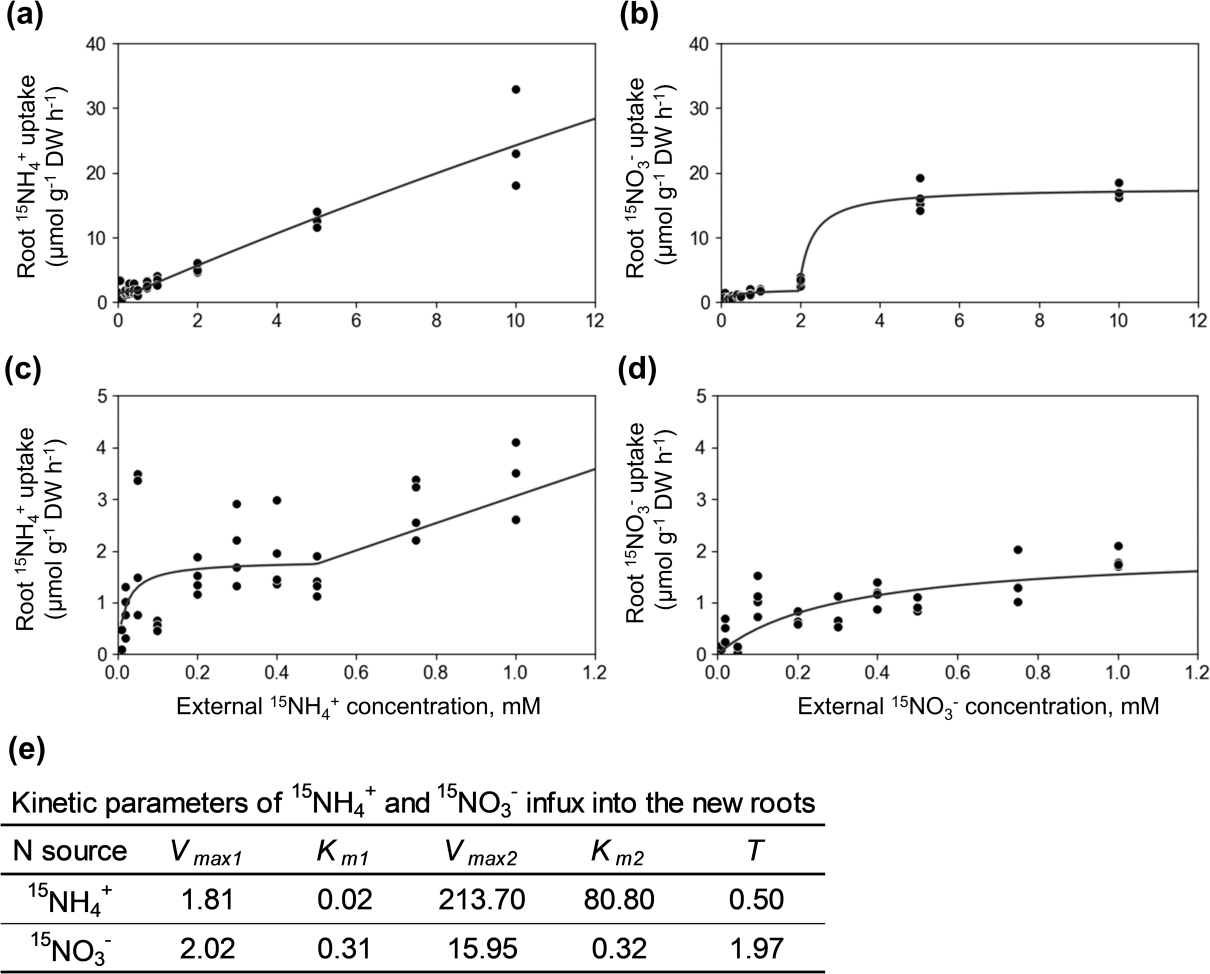
Estimation of the concentration ranges of low‐ and high‐affinity transporters using the two‐stage Michaelis–Menten equation for ^15^NH_4_^+^ and ^15^NO_3_^−^ influx into the new roots of tea plants. (a) ^15^NH_4_^+^ uptake by roots in the concentration range 0–10 mM. (b) ^15^NO_3_^−^ uptake by roots in the concentration range 0–10 mM. (c) and (d) Enlarged images of low ^15^NH_4_^+^ and ^15^NO_3_^−^ concentrations (0–1.0 mM), respectively. (e) *Vmax*, *Km*, and *T* values of ^15^NH_4_^+^ and ^15^NO_3_^−^ in new roots.

The influx of ^15^NH_4_^+^ in tea roots followed a typical biphasic pattern. At low concentrations (< 0.5 mM), the influx of ^15^NH_4_^+^ was in accordance with a Michaelis–Menten curve. The K_m_ value was 0.02 mM and V_max_ was 1.81 μmol/g (DW, 1 h) (Figure 2a, c, e). At high concentrations (≥ 0.5 mM), the influx of ^15^NH_4_^+^ increased almost linearly as the ^15^NH_4_^+^ concentration increased. The K_m_ value was 81.3 mM and V_max_ was 213.7 μmol/g (DW, 1 h). Similarly, the influx of ^15^NO_3_^−^ was investigated. The influx of ^15^NO_3_^−^ was consistent with a typical Michaelis–Menten curve for concentrations up to 2 mM (Figure 2b, d, e). The K_m_ value was 0.31 mM and V_max_ was 2.02 μmol/g (DW, 1 h). The influx of ^15^NO_3_^−^ also increased and plateaued (i.e., a curve) at concentrations exceeding 2 mM. The K_m_ value was 2.29 mM and V_max_ was 16.0 μmol/g (DW, 1 h).

### 3.3 Effect of Inorganic N Sources on the N Concentration and Content of Tea Plants

**FIGURE 3:**
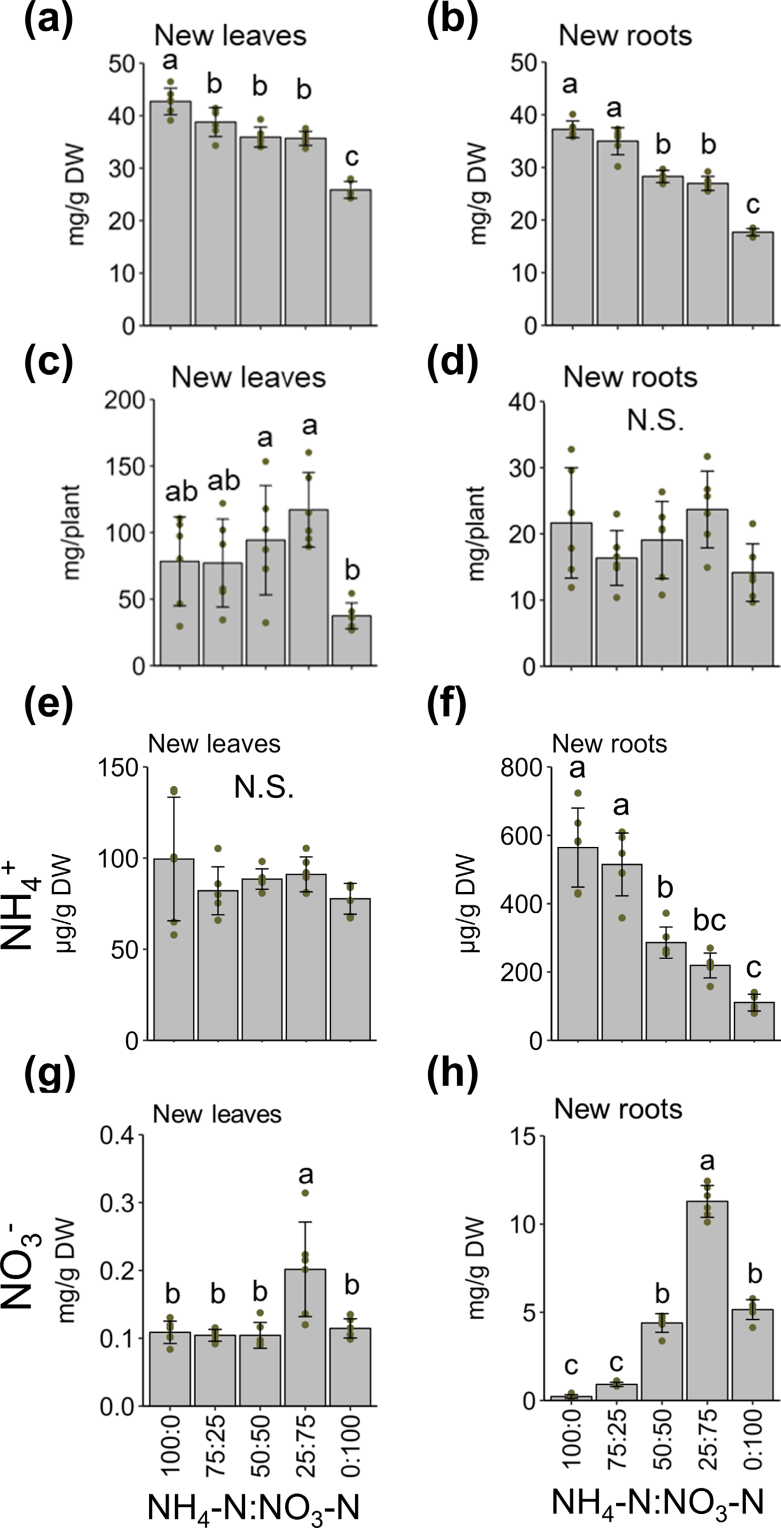
Effect of inorganic N source ratios on total N, ammonium, and nitrate concentrations in tea plants. (a, b) N concentration in tea plants: (a) new leaves and (b) new roots. (c, d) N content in tea plants: (c) new leaves and (d) new roots. (e, f) Ammonium concentration in tea plants: (e) new leaves and (f) new roots. (g, h) Nitrate concentration in tea plants: (g) new leaves and (h) new roots. Data are presented as the mean ± SD Dots represent replicates (n = 6). Different letters indicate significant differences as determined by Tukey's HSD test (*p* < 0.05). N.S., not significant (*p* ≥ 0.05).

The total N concentration in new leaves and new roots tended to increase as the proportion of ammonium increased (Figure 3a, b). The total N content in new leaves and new roots peaked at ammonium:nitrate ratio of 25:75 (Figure 3c, d). Subsequently, the ammonium and nitrate concentrations were measured. The ammonium concentration of new leaves did not differ significantly between treatments (Figure 3e). The ammonium concentration in new roots tended to increase as the proportion of ammonium increased (Figure 3f). The nitrate concentration of new leaves was highest at an ammonium:nitrate ratio of 25:75, with no significant differences among the effects of the other treatments (Figure 3g). The nitrate concentration in new roots increased as the proportion of nitrate increased, with peak levels when the ammonium:nitrate ratio was 25:75, but further increases (i.e., ammonium:nitrate ratio of 0:100) caused the nitrate concentration in new roots to decrease (Figure 3h).

### 3.4 Effect of Inorganic N Sources on the FAA Concentration of Tea Plants

**FIGURE 4:**
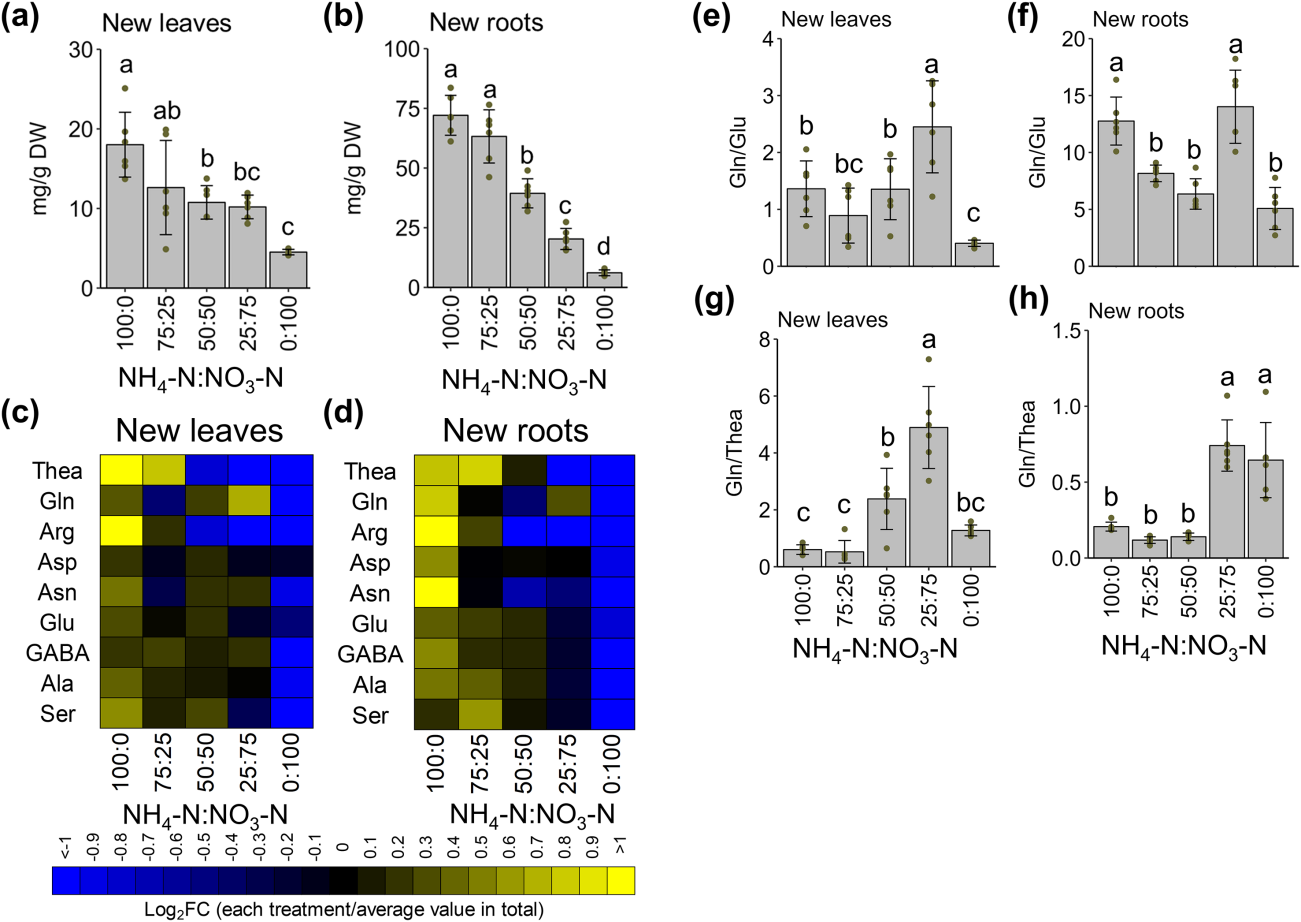
Effect of inorganic N source ratios on FAA concentrations in tea plants. (a, b) Total free amino acid concentration in tea plants: (a) new leaves and (b) new roots. (c, d) Heat map of free amino acid concentrations in tea plants. The color spectrum from yellow to blue corresponds to the relative concentration of each amino acid. The fold‐change (FC) value for each amino acid was calculated by dividing the amino acid concentration of a sample by the mean concentration for five treatments. Log_2_FC denotes the base‐2 logarithm of FC: (c) new leaves and (d) new roots. (e, f) Gln:Glu ratio in new leaves (e) and new roots (f). (g, h) Gln:Thea ratio in new leaves (g) and new roots (h). Data are presented as the mean ± SD Dots represent replicates (n = 6). Different letters indicate significant differences as determined by Tukey's HSD test (*p* < 0.05).

The total FAA concentration increased in both new leaves and new roots as the proportion of ammonium increased (Figure 4a, b). The concentrations of individual FAAs (data from Supplementary Table 1) were compared between treatments in heat maps (Figure 4c, d). Most FAA concentrations in new leaves and new roots increased as the proportion of ammonium increased. In particular, large changes were observed for theanine and arginine. There was no difference in the theanine concentration between the treatments with ammonium:nitrate ratios of 100:0 and 75:25, but the arginine concentration differed significantly (Supporting Figure 2). The arginine concentration was affected more by the external ammonium concentration than the theanine concentration. The glutamine concentration changes differed from the changes in the concentrations of the other FAAs. Interestingly, the glutamine concentration in new leaves was highest at an ammonium:nitrate ratio of 25:75 (Figure 4c). This treatment also resulted in the second‐highest glutamine concentration in new roots (Figure 4d).

The glutamine/glutamate (Gln/Glu) values for new leaves and new roots were higher at an ammonium:nitrate ratio of 100:0 than at 0:100 (Figure 4e, f), suggesting that GS activity exceeded the GOGAT activity capacity and resulted in excessive glutamine at an ammonium:nitrate ratio of 100:0. Notably, the Gln/Glu values for new leaves and new roots were highest at an ammonium:nitrate ratio of 25:75, suggesting that this treatment caused GS activity to exceed the GOGAT activity capacity, resulting in excessive glutamine production. The glutamine/theanine (Gln/Thea) values for new leaves and new roots were lower at an ammonium:nitrate ratio of 100:0 than at 0:100. This suggests that TS was more active than GS at 100:0, thereby increasing the production of theanine from glutamate. In new leaves, the Gln/Thea value was high at ammonium:nitrate ratios of 50:50 and 25:75 (Figure 4g), implying that the amount of theanine transported from the roots to the shoots decreased. Treatments with ammonium:nitrate ratios of 25:75 and 0:100 resulted in the highest Gln/Thea values for new roots (Figure 4h). This may indicate glutamine synthesis is prioritized over theanine synthesis under high nitrate conditions.

### 3.5 Effect of Inorganic N Sources on the Expression of N Metabolism Genes in Tea Plants

**FIGURE 5:**
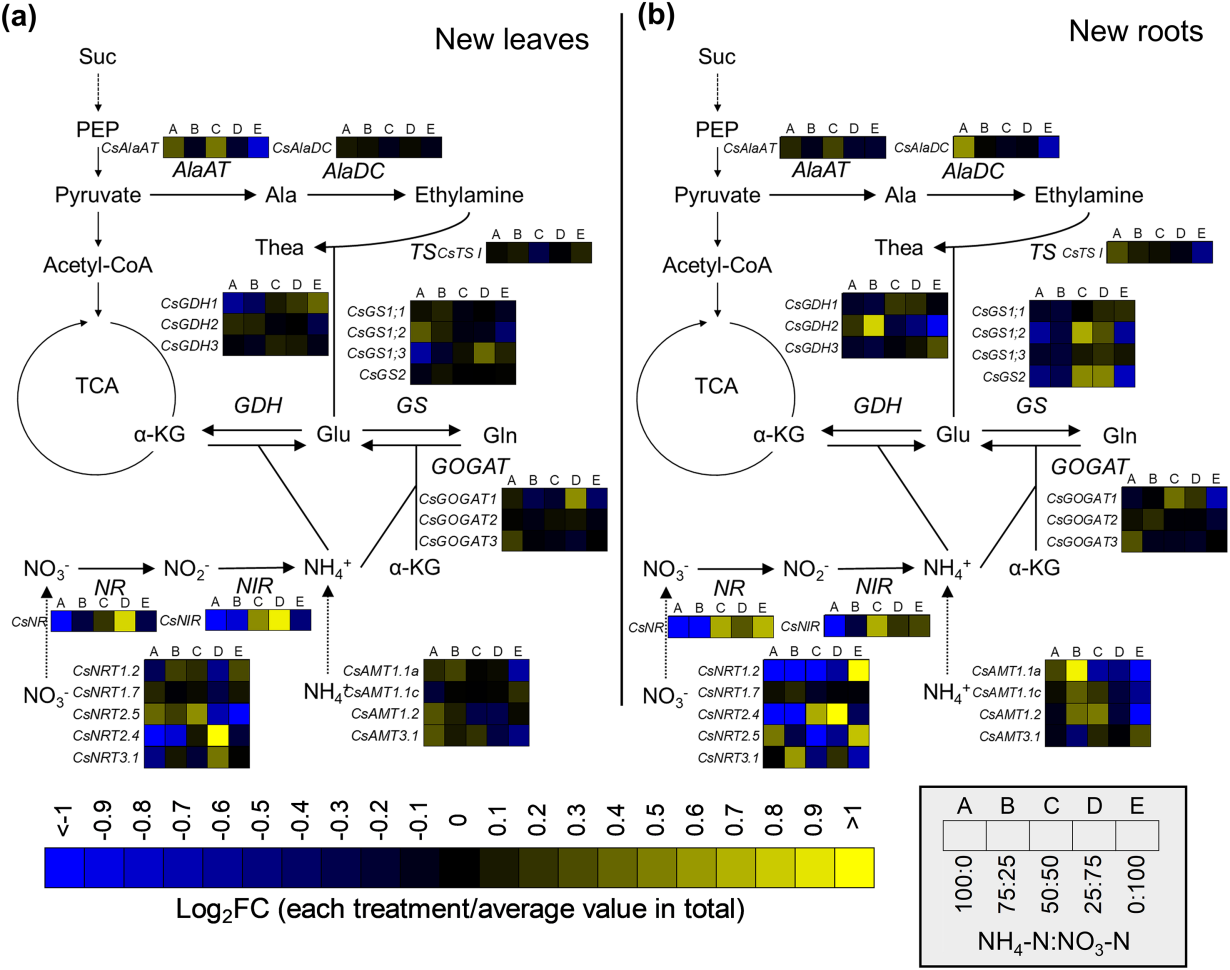
Expression profiles of genes involved in N metabolism in response to inorganic N source ratios in tea plants. NRT, nitrate transporter; AMT, ammonium transporter; NR, nitrate reductase; NIR, nitrite reductase; GS, glutamine synthase; GOGAT, glutamate synthase; GDH, glutamate dehydrogenase; TS, theanine synthetase; AlaDC, alanine decarboxylase; AlaAT, alanine aminotransferase; Suc, sucrose; PEP, phosphoenol pyruvic acid; TCA, tricarboxylic acid cycle; α‐KG, α‐ketoglutaric acid; Ala, alanine; Thea, theanine; Glu, glutamate; Gln, glutamine. (a, b) Fold‐change (FC) in the expression of genes related to N metabolism in new leaves and new roots following treatments with different ammonium:nitrate ratios. The color spectrum from yellow to blue corresponds to the relative expression of each gene. The FC value for each gene was calculated by dividing the gene expression level of a sample by the mean expression level for five treatments. Log_2_FC denotes the base‐2 logarithm of FC: (a) new leaves and (b) new roots.

Differences in inorganic N sources affect N metabolite concentrations. Thus, an RNA‐seq analysis was performed to compare N metabolism gene expression levels. A hierarchical clustering analysis of the RNA‐seq data revealed gene expression differences among treatments (Supporting Figure 3). In particular, ammonium:nitrate ratios of 100:0 and 75:25 were mostly separated from ammonium:nitrate ratios of 25:75 and 0:100 in different clusters. The expression levels of N metabolism genes (data from Supplementary Table 2) were compared between treatments in heat maps (Figure 5). The analyzed N metabolism genes were described previously (Wang et al. 2022; Zhang et al. 2023). The differences in nitrate and ammonium transporter gene expression levels among treatments were greater for new roots than for new leaves (Figure 5a, b). The *CsNRT2.4* expression level in new leaves was significantly higher following the treatment with ammonium:nitrate ratio of 25:75 than after the other treatments (Supporting Figure 4). In new roots, *CsNRT2.4* was expressed at much higher levels than the other nitrate transporter genes (Supporting Figure 5). *CsNRT2.4* expression increased as the proportion of nitrate increased, but decreased when the ammonium:nitrate ratio was 0:100 (Figure 5b). Ammonium transporter genes tended to be expressed at high levels when the proportion of ammonium was 50% or higher (i.e., ammonium:nitrate ratios of 50:50, 75:25, and 100:0). Among the examined genes encoding metabolic enzymes, *CsNR* and *CsNIR* were most highly expressed at an ammonium:nitrate ratio of 25:75 (Figure 5a), which was consistent with the effects of this treatment on growth. In terms of *CsNR*, its expression level was significantly higher at an ammonium:nitrate ratio of 25:75 than at the other tested ammonium:nitrate ratios (Supporting Figure 4). In new roots, *CsNR* and *CsNIR* expression levels were higher at ammonium:nitrate ratios of 25:75, 50:50, and 0:100 than at ammonium:nitrate ratios of 100:0 and 75:25 (Figure 5b). The expression levels of *CsTS I* and *CsAlaDC*, which are involved in theanine synthesis, increased in new roots as the proportion of ammonium increased. The expression of both *CsTS I* and *CsAlaDC* differed significantly between the treatments with ammonium:nitrate ratios of 100:0 and 0:100 (Supporting Figure 5). *CsGS1;2*, *CsGS2*, and *CsGOGAT1* expression levels in new roots tended to be highest at ammonium:nitrate ratios of 50:50 and 25:75.

### 3.6 Effect of Inorganic N Sources on the Expression of Transcription Factor and Kinase Genes Regulating N Metabolism

The expression levels of genes encoding transcription factors that regulate the expression of N metabolism genes as well as kinase genes were investigated using RNA‐seq data for new leaves and new roots. The top 10 genes in terms of homology to Arabidopsis or rice sequences (score >200) in the Tea Plant Information Archive database (http://tpia.teaplants.cn/index.html) were extracted (Supplementary Tables 3–8). We searched for tea proteins highly homologous to AtNIGT1.1, AtbZIP1, AtSTOP1, AtTCP20, and AtTGA1, which are transcription factors that regulate the expression of nitrate transporter genes and nitrate assimilation‐related genes in Arabidopsis. A phylogenetic tree was constructed to identify Arabidopsis proteins closely related to the corresponding proteins in tea. Three CsNIGT1s, one CsTGA, two CsTCP20s, and eight STOPs closely related to Arabidopsis proteins were identified (Supporting Figures 6–9). By contrast, a protein highly homologous to AtbZIP1 was not detected. In rice, we identified six genes encoding proteins highly homologous to OsACTPK1 (Supporting Figure 10), which suppresses ammonium transporter activities (Beier et al. 2018). The expression levels of the identified tea genes were analyzed by RNA‐seq (Supplementary Table 8). We also investigated the expression levels of *CsNLP* genes, which reportedly encode proteins that activate the expression of N metabolism genes in tea (Li et al. 2024), *CsLBD37*, *38*, and *39*, which suppress the expression of N metabolism‐related genes in tea (Teng et al. 2022), and *CsMYB78*, which suppresses *CsGS* expression (Wen et al. 2020). The expression levels of *CsMYB6*, *CsHHO3*, and *CsMYB40*, which encode regulators of genes in the theanine synthesis pathway, were also examined (Zhang et al. 2021b; Guo et al. 2022), but RNA‐seq data for these genes were unavailable in this study, possibly because these genes were expressed at low levels.

**FIGURE 6:**
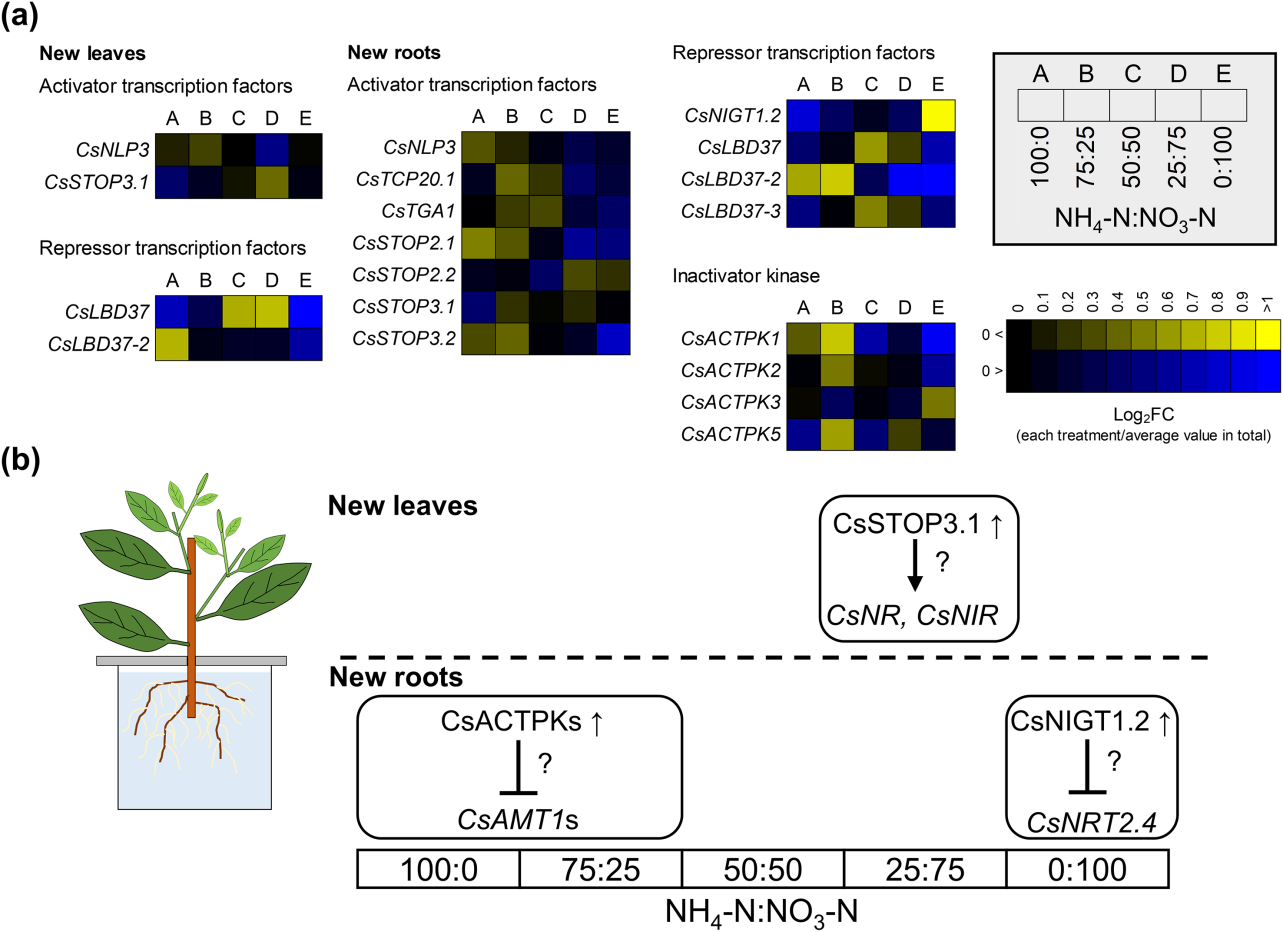
Expression profiles of regulatory genes involved in N metabolism in response to inorganic N source ratios in tea plants. (a, b) Fold‐change (FC) in gene expression in new leaves and new roots. The presented genes were extracted from Supplementary Table 9 (maximum expression level/minimum expression level > 1.5 and significant differences among treatments). The color spectrum from yellow to blue corresponds to the relative expression of each gene. The FC value for each gene was calculated by dividing the gene expression level of a sample by the mean expression level for five treatments. Log_2_FC denotes the base‐2 logarithm of FC. (b) Diagram of the regulatory system.

In new leaves, the expression level of *CsSTOP3.1*, which has regulatory effects on *NR* expression in Arabidopsis (Tokizawa et al. 2023), was correlated with *CsNR* and *CsNIR* expression levels (Figures 5a and 6a). In new roots, *CsACTPK1* expression tended to increase at ammonium:nitrate ratios of 100:0 and 75:25 (relative to the expression induced by the other treatments) (Figure 6a). In Arabidopsis, AtNIGT1s inhibit nitrate transporter gene expression (Maeda et al. 2018). In the current study, *CsNIGT1.2* expression increased after the treatment with an ammonium:nitrate ratio of 0:100 (Figure 6a). We speculated that tea also has a mechanism that restricts uptake by new roots when external ammonium and nitrate concentrations are high (Figure 6b).

### 3.7 Changes in Gene Expression in new Leaves Under Different N Conditions

**FIGURE 7:**
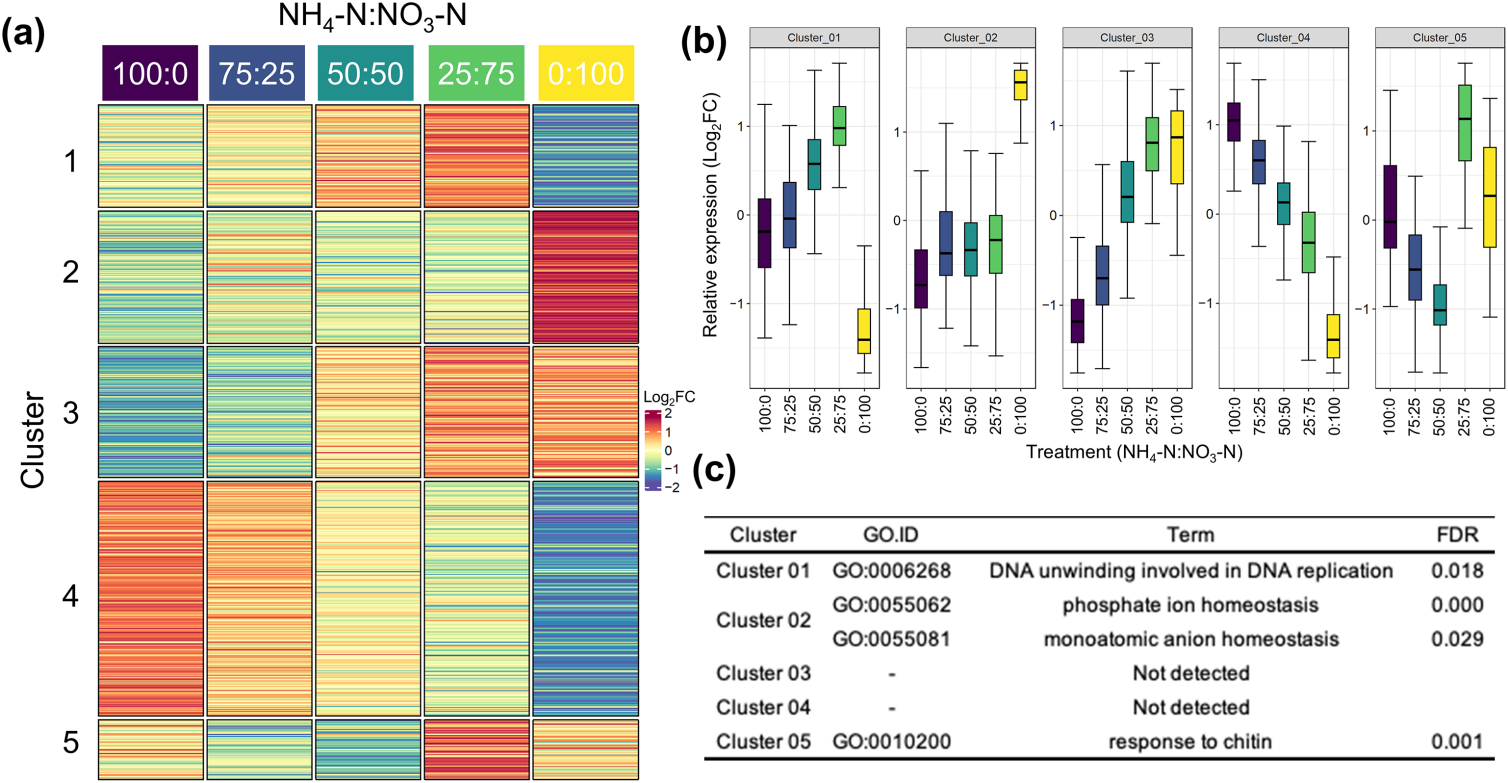
Gene expression profiles and Gene Ontology analysis of new leaves. (a) Transcript levels in tea plants exposed to five different conditions were determined via an RNA‐seq analysis. The fold‐change (FC) value for each gene was calculated by dividing the gene expression level of a sample by the mean expression level for five treatments. Log_2_FC denotes the base‐2 logarithm of FC. A non‐hierarchical K‐means clustering (K = 5) analysis was performed. The color spectrum from red to blue corresponds to the relative expression of each gene. (b) Average expression levels of genes in each cluster reveal five distinct expression patterns. Transcriptome order is in accordance with that in Figure 6a. (c) Significantly enriched [false discovery rate (FDR) < 0.05] Gene Ontology (GO) categories (biological function) of each gene cluster.

We investigated the effects of cultivation conditions that differ in terms of inorganic N sources on gene expression. The examined genes were classified into five groups according to the K‐means method (Figure 7a). There were groups comprising genes with expression levels that increased as the proportion of nitrate increased as well as groups consisting of genes with expression levels that increased as the proportion of ammonium increased (Figure 7b). Similar to the growth of aerial plant parts, the expression levels of gene groups changed the most following the treatment with an ammonium:nitrate ratio of 25:75. A GO analysis revealed Cluster I was enriched with genes related to DNA synthesis (GO:0006268; DNA unwinding involved in DNA replication) (Figure 7c). This GO term was assigned to three *mini‐chromosome maintenance* (*MCM*) genes (Supplementary Table 16). MCM proteins serve as licensing factors for DNA replication, thereby ensuring genomic DNA is replicated completely (Tuteja et al. 2011). Thus, increased expression of genes related to DNA synthesis may promote new leaf growth.

### 3.8 Identification of Genes Influencing Tea Phenotypes via Transcriptome‐Based Regression Modeling

**FIGURE 8:**
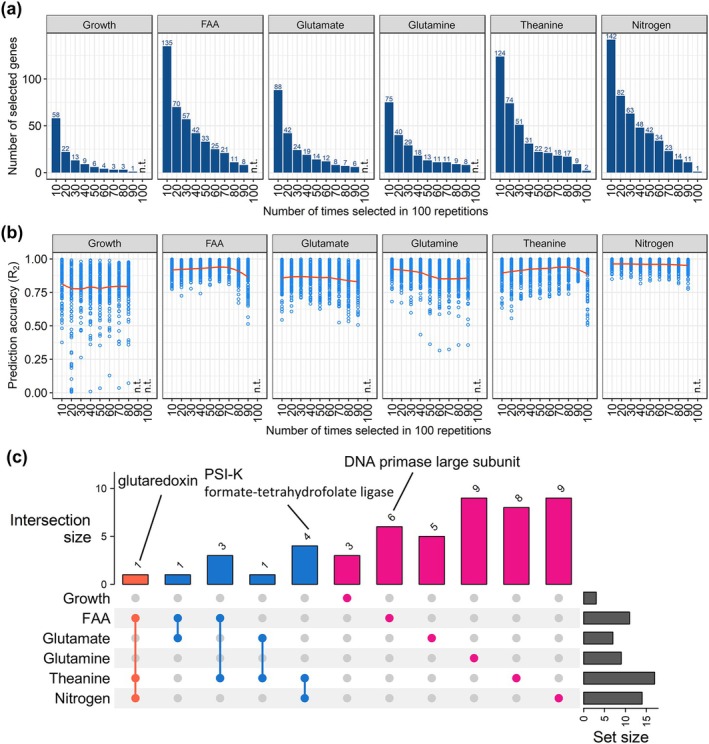
Feature selection and transcriptome‐based modeling performance. Growth, new leaf growth; FAA, total free amino acid concentration; Nitrogen, total N concentration. FAA, glutamate, glutamine, theanine, and N concentrations in new leaves were used. (a) Transcriptome data for new leaves were used for the analysis. The number of genes selected by the Boruta feature selection algorithm is indicated. Genes selected 10–100 times (at intervals of 10) in 100 replicates for each phenotype are presented. (b) Prediction accuracy for the transcriptome‐based modeling involving the Boruta‐selected genes. Plots show the results for each replicate. Red curves represent locally estimated scatterplot smoothing (LOESS) lines. (c) UpSet plot of the genes selected 80 times (out of 100 times) for each phenotype. n.t., not tested.

To estimate the extent to which the transcriptome of new leaves can explain phenotypic variations in new leaf growth and FAA, glutamate, glutamine, theanine, and N concentrations in response to treatments with different inorganic N application ratios, a transcriptome‐based regression model was constructed for tea plant phenotypes. First, the Boruta feature selection algorithm was used to eliminate non‐informative transcriptome data (Figure 8a). Three genes extracted more than 80 times (out of 100 times) for leaf growth explained approximately 80% of the growth variation due to inorganic N conditions (Figure 8b). For total FAA and theanine, selecting genes extracted more than 80 times decreased the prediction accuracy. A total of 7–17 genes for total FAA, glutamate, glutamine, theanine, and total N concentrations extracted more than 80 times (out of 100 times) explained approximately 80%–95% of the phenotypic variation. The prediction accuracy was highest for total N, with the expression patterns of 14 genes explaining most of the variation. Among the commonly extracted genes, one gene that was commonly extracted for FAA, theanine, and N encoded glutaredoxin, which is involved in redox reactions (Figure 8c). Four genes were commonly extracted for theanine and N, including genes encoding PSI‐K, a subunit of photosystem I, and tetrahydrofolate ligase. The gene extracted for leaf growth encoded a DNA primase large subunit involved in DNA replication (Supplementary Table 17).

The importance and partial dependence of variables in the regression model constructed using a random forest algorithm enabled the interpretation of the nonlinear relationship between phenotypes and gene expression levels (Supporting Figure 11). The partial dependence of each phenotype gradually increased or decreased as gene expression levels increased. Partial dependence for the glutaredoxin gene gradually decreased, whereas partial dependence for the genes encoding PKS‐I, tetrahydrofolate ligase, and the DNA primase large subunit gradually increased.

## 4 Discussion

N metabolism is a major factor influencing tea plant growth and quality. In this study, we evaluated the effects of fertilizing with different ammonium:nitrate ratios on tea plants. The N concentration and content were correlated with the FAA content and growth, respectively (Figure 1d, h, 3a–d, 4a, b). For aerial plant parts, treatments with both ammonium and nitrate resulted in better growth than treatments with either of the two N sources applied alone, with optimal growth observed for the treatment with an ammonium:nitrate ratio of 25:75. The total FAA concentration tended to be high when the proportion of ammonium was high. Unlike the concentrations of some FAAs (e.g., theanine and arginine), the glutamine concentration in new leaves peaked at an ammonium:nitrate ratio of 25:75. Moreover, the gene expression data for new roots indicated that *CsAlaDC* and *CsTS I* expression levels increased as the proportion of ammonium increased, while *CsGS1;2* and *CsGS2* expression peaked at an ammonium:nitrate ratio of 25:75. Accordingly, changes to the ammonium:nitrate ratio can alter theanine and glutamine biosynthesis.

Ishigaki (1978) reported that co‐fertilization with ammonium and nitrate promotes tea plant growth, but the associated molecular mechanism was unclear because of a lack of genomic research. In the present study, a transcriptome analysis was performed, which revealed *CsGS1;2* and *CsGS2* expression levels in new roots were highest at ammonium:nitrate ratios of 50:50 and 25:75, which were also conducive to growth. By contrast, *CsAlaDC* and *CsTS I* expression levels were lower following the treatments with ammonium:nitrate ratios of 50:50 and 25:75 than after the treatments with ammonium:nitrate ratios of 100:0 and 75:25. These results suggest that the GS activity: TS activity ratio is higher at ammonium:nitrate ratios of 50:50 and 25:75 than at 100:0 and 75:25. In fact, Gln/Thea values were higher at ammonium:nitrate ratios of 50:50 and 25:75 than at 100:0 and 75:25 (Figure 4g).

A cluster analysis of new leaves showed that Cluster 1 gene expression patterns were similar to the trends in new leaf growth (Figures 1d and 7b). At an ammonium:nitrate ratio of 25:75, the glutamine concentration increased in new leaves (Figure 4c). In plants, glutamine promotes growth (Franklin et al. 2017), while also serving as a signaling molecule (Foyer et al. 2003). In the current study, an increase in the endogenous glutamine concentration may have enhanced new leaf growth. According to animal studies, glutamine is an amino acid that serves as a source of carbon and N and supports nucleotide biosynthesis via signal transduction (Yoo et al. 2020; Tong et al. 2009). Ishigaki (1978) reported that tea plants grow poorly when the proportion of nitrate exceeds 50%, but in the current study, the highest growth was observed at an ammonium:nitrate ratio of 25:75. This discrepancy between studies may be due to differences in other nutrients and pH. Hence, future studies will need to examine other nutrients and N utilization.

We speculated that the accumulation of glutamine in new leaves positively affected growth. However, the accumulation of glutamine at an ammonium:nitrate ratio of 100:0 was comparable to that at 50:50, but the growth at 100:0 was lower than that at 50:50. This may be related to the accumulation of theanine, which is affected by abiotic stresses (e.g., salinity) (Chen et al. 2021). The expression level of *CsACTPK1*, which encodes a protein that inactivates ammonium transporters in tea, tended to be high at ammonium:nitrate ratios of 100:0 and 75:25 (Figure 6a). A hydroponic solution with an ammonium concentration exceeding 30 ppm (2.14 mM) may be stressful for tea. To assess this possibility, tests involving different ammonium:nitrate ratios and changing N concentrations will need to be conducted. Treatments with only ammonium or nitrate promoted growth less than treatments with both N sources. There may be multiple reasons for this observation. There were significant differences in FAA accumulation (Figure 4). When nitrate alone was applied, tea plants absorbed nitrate, but the subsequent reduction of nitrate and nitrite may not have occurred. Ammonium and nitrate uptake rates were similar when the N concentration was 2.86 mM (Figure 2). In new leaves, *CsNR* and *CsNIR* expression levels were lower when the ammonium:nitrate ratio was 0:100 than when the ratio was 25:75 (Figure 5a). There may be other reasons for the limited growth when nitrate alone was applied. Transcriptome results showed that there were genes that were activated under nitrate‐only conditions. In addition, a GO analysis indicated Cluster 2 genes were related to phosphate ion homeostasis (Figure 7c). This GO term was assigned to two glycerophosphodiester phosphodiesterase genes and a purple acid phosphatase gene (Supplementary Table 16). Glycerophosphodiester phosphodiesterase genes are responsive to phosphorus deficiency and encode enzymes that maintain phosphorus concentration homeostasis (Mehra et al. 2019; J. Wang, Pan, et al. 2021). Thus, nitrate‐only conditions may be associated with phosphorus deficiency, but this will need to be verified by a more thorough analysis of elemental concentrations.

The expression of *CsNR* and *CsNIR* genes in the N metabolism‐related pathway changed substantially depending on inorganic N proportions (Figure 5). In new roots, *CsNR* was highly expressed at ammonium:nitrate ratios of 50:50 and 0:100. The *CsNIR* expression level was lower at 0:100 than at 50:50. When nitrate is applied alone (0:100), nitrite may accumulate and inhibit growth. *CsNR* and *CsNIR* expression levels in new leaves were correlated with the nitrate concentration in new roots (Figures 3h and 7a). In rice, high NR activities lead to increased grain yield (Gao et al. 2019). High *CsNR* and *CsNIR* expression levels in new leaves may enhance growth (Figures 1d and 5a).

A transcriptome analysis clarified the changes in gene expression and metabolite concentrations. Nitrate concentrations in new roots were highly correlated with the expression of *CsNRT2.4*, which is a key gene for nitrate uptake by tea plant roots (Zhang et al. 2021a). In addition, *CsNRT2.4* expression suppressed by CsNIGT1.2 was predicted at an ammonium:nitrate ratio of 0:100 (Figures 5b and 6). In Arabidopsis, AtNIGT1s suppress the expression of nitrate transporter genes when nitrate concentrations are high (Maeda et al. 2018). Excessive nitrate accumulation was not detected in new leaves when the nitrate proportion was high (ammonium:nitrate ratios of 25:75 and 0:100) (Figure 3g). The expression levels of *CsNR* and *CsNIR*, which are related to nitrate reduction, in new leaves were correlated with the nitrate concentration of new roots (Figure 5a). In pakchoi, cultivars that accumulate nitrate at low levels reportedly have higher nitrate reductase activities than cultivars that accumulate relatively large amounts of nitrate (Luo et al. 2006). Nitrate did not accumulate excessively in new leaves possibly because of changes in *CsNR* and *CsNIR* expression levels, but this possibility will need to be experimentally verified. In Arabidopsis, AtSTOP1 regulates *AtNRT1.1* and *AtNR1* expression (Tokizawa et al. 2023). In the present study, eight CsSTOPs were identified (Supporting Figure 8), three of which were highly homologous to AtSTOP1, whereas another three were highly homologous to AtSTOP2. The remaining two CsSTOPs were named CsSTOP3.1 and CsSTOP3.2. Notably, the *CsSTOP3.1* expression pattern in new leaves was similar to *CsNR* and *CsNIR* expression patterns in new leaves, suggesting *CsNR* and *CsNIR* expression in new leaves may be regulated by CsSTOP3.1. Changes in *CsNLP* expression levels did not explain the changes in *CsNRT*, *CsNR*, and *CsNIR* expression levels probably because CsNLPs regulate transcription through post‐translational activation rather than through expression level changes. In Arabidopsis, AtNLP activities are also post‐translationally regulated by nitrate signals (Liu et al. 2017; Liu et al. 2022). Additional research on transcription factors in tea may further characterize metabolic changes in response to environmental conditions (e.g., N nutrient availability).

Our transcriptome modeling may have captured molecules not yet confirmed to be involved in new leaf growth and changes in N metabolites. Among the genes selected for the transcriptome modeling of leaf growth was a gene encoding the DNA primase large subunit (Figure 8c). Genes involved in DNA replication were also extracted using the K‐means method (Supplementary Table 16). The increased growth of new leaves may be related to increased cell proliferation. The genes selected for the modeling of theanine and N contents in new leaves included genes involved in antioxidant activities, such as genes encoding glutaredoxin, superoxide dismutase, and thioredoxin domain‐containing protein, as well as several genes encoding chlorophyll *a*/*b*‐binding proteins (Supplementary Table 17). The expression of antioxidant activity‐related genes decreased as theanine and N concentrations increased. Considering theanine activates the scavenging of reactive oxygen species (Wang et al. 2025), high theanine concentrations may have decreased the expression of antioxidant genes. The expression of several genes encoding chlorophyll *a*/*b*‐binding proteins increased as the proportion of ammonium increased. This may be related to the reported increase in photosynthesis following increases in the N content (Sinclair and Horie 1989).

An analysis of ammonium and nitrate absorption kinetics provided insights into the effects of experimental conditions on tea plants. For tea plant roots, the ammonium absorption rate was higher than the nitrate absorption rate at concentrations less than 1 mM, which is consistent with previous findings (Yang et al. 2013). At concentrations greater than 2 mM, the ammonium absorption rate increased linearly, but the nitrate absorption rate almost plateaued at 5 mM. In an earlier study by Yang et al. (2013), the absorption rates of both ammonium and nitrate increased linearly at concentrations exceeding 1 mM. This difference in study results may be caused by the differences in the tea plant growth stages that were analyzed. Specifically, Yang et al. (2013) used 2‐month‐old seedlings, whereas we used 4‐month‐old tea plants derived from 1‐year‐old rooted tea cuttings. The nitrate absorption rate reportedly varies among growth stages (Cui et al. 2017). In tea plants, ammonium absorption also changes before and after budding (Okano et al. 1994). The effects of tea plant growth stages on the nitrate absorption rate will need to be determined.

This study provides new insights relevant to N fertilization management for tea cultivation. The accumulation of FAAs and theanine affects tea leaf quality. Because of the trade‐off between quality and yield, balancing the application of N sources will depend on what is prioritized. In tea fields with conventional fertilizer applications, more than 50% of the N in the soil is ammonium from March to June, which is when the crop is harvested, whereas most of the N in the soil is nitrate from November to February (Hirono and Nonaka 2014). In addition, the distribution of ammonium and nitrate differs depending on soil depth (Mukasa et al. 1973). The upper soil layer has a higher proportion of ammonium, while the lower soil layer has a higher proportion of nitrate. This is not because of a change in nitrate concentrations. Instead, it is because the applied ammonium is maintained with almost no movement or is transformed into nitrate and lost. Therefore, to repeat this study under field conditions, a marker that can determine how plants recognize the inorganic N ratio status should ideally be developed.

Tea leaf growth and quality change in response to inorganic N fertilization. In future studies, we will need to find ways to control tea fields to optimize fertilizer applications. More specifically, the relationship between the inorganic N status of tea fields and tea plant quality and growth should be explored.

## Author Contributions

*Conceptualization*: Takuo Enomoto, Natsuki Tone, Yuhei Hirono, Hiroto Yamashita, and Takashi Ikka. *Data curation*: Takuo Enomoto. *Formal analysis*: Takuo Enomoto, Natsuki Tone, Takaya Ishii, and Hiroto Yamashita. *Funding acquisition*: Takuo Enomoto and Yuhei Hirono. *Investigation*: Takuo Enomoto, Natsuki Tone, Takaya Ishii, Hisako Hirono, and Ayako Oi. *Methodology*: Takuo Enomoto, Takaya Ishii, and Yuhei Hirono. *Project administration*: Takuo Enomoto and Hiroto Yamashita. *Supervision*: Yuhei Hirono and Takashi Ikka. *Writing – original draft*: Takuo Enomoto. *Writing–review and editing*: Takuo Enomoto, Natsuki Tone, Yuhei Hirono, and Hiroto Yamashita. All authors have read and agreed to the published version of this manuscript.

## Conflicts of Interest

The authors have no conflicts of interest to declare.

## Peer Review

The peer review history for this article is available in the Supporting Information for this article.

## Supporting information


**Data S1** Supplementary Peer Review.

**Table S1** NH4‐N:NO3‐N ratio at 100:0.
**Table S2** NH4‐N:NO3‐N ratio at 75:25.
**Table S3** NH4‐N:NO3‐N ratio at 50:50.
**Table S4** NH4‐N:NO3‐N ratio at 25:75.
**Table S5** NH4‐N:NO3‐N ratio at 0:100.
**Table S6** Hydoroponic solution for growth conditions.
**TableS7** All value of FAAs. Values were concentration or ratio for each condition (NH4‐N:NO3‐N, A; 100:0, B; 75:25, C; 50:50, D;25:75, E; 0:100).
**Table S8** All conditions expression of N‐metabolism genes in new leaves and new roots. Expression values were count per million for each condition (NH4‐N:NO3‐N, A; 100:0, B; 75:25, C; 50:50, D;25:75, E; 0:100).
**Table S9** List of NIGT genes and amino acid sequences.
**Table S10** List of STOP genes and amino acid sequences.
**Table S11** List of TCP genes and amino acid sequences.
**Table S12** List of TGA genes and amino acid sequences.
**Table S13** List of ACTPK genes and amino acid sequences.
**Table S14** All conditions gene expression of N‐metabolism regulated genes in new leaves and new roots. Expression values were count per million for each condition (NH4‐N:NO3‐N, A; 100:0, B; 75:25, C; 50:50, D;25:75, E; 0:100). N.D., not ditected.
**Table S15** Average gene expression of N‐metabolism regulated genes in new leaves and new roots. Expression values were averages for each condition (NH4‐N:NO3‐N, A; 100:0, B; 75:25, C; 50:50, D;25:75, E; 0:100). N.D., not ditected. Different letters indicate significant differences as determined using Tukey’s HSD test (*p* < 0.05). S., significant (*p* < 0.05). N.S., not significant (*p* > 0.05).
**Table S16** GO terms assigned to genes. Genes annotated with the GO terms in Figure 7c are listed.
**Table S17** Genes selected by the Boruta feature selection algorithm. These genes were extracted more than 80 times (out of 100 times) by the Boruta feature selection algorithm (Figure 8a). Common genes were extracted from the UpSet plot (Figure 8c).

**Figure S1** **Flow chart of the influx kinetics experiment.**
**Figure S2 Effect of inorganic N sources ratio on amino acid concentration in tea plants.** (a, b) Free amino acids concentration in tea plants: (a) new leaves, (b) new roots. Data and error bars are the mean ± SD Plots are replicates (*n* = 6). Different letters indicate significant differences as determined using Tukey’s HSD test (*p* < 0.05).
Figure S3 Hierarchical clustering analysis of new leaves and new roots.
**Figure S4 Gene expression of N‐metabolism genes in new leaves.** Expression level of N‐metabolism genes in new leaves were shown. Data and error bars are the mean ± SD. Plots are replicates (*n* = 6). Different letters indicate significant differences as determined using Tukey’s HSD test (*p* < 0.05). N.S., not significant (*p* ≥ 0.05).
**Figure S5 Gene expression of N‐metabolism genes in new roots.** Expression level of N‐metabolism genes in new roots were shown. Data and error bars are the mean ± SD. Plots are replicates (*n* = 6). Different letters indicate significant differences as determined using Tukey’s HSD test (*p* < 0.05). N.S., not significant (*p* ≥ 0.05).
**Figure S6 Phylogenetic tree of NIGTs from *Arabidopsis thaliana* and *Camellia sinensis* .** Protein sequences were aligned by MEGA11.0, and the tree was constructed by MEGA11.0 using the Neighbor‐Joining method.
**Figure S7 Phylogenetic tree of STOPs from *A. thaliana* and *C. sinensis* .** Protein sequences were aligned by MEGA11.0, and the tree was constructed by MEGA11.0 using the Neighbor‐Joining method.
**Figure S8 Phylogenetic tree of TCPs from *A. thaliana* and *C. sinensis* .** Protein sequences were aligned by MEGA11.0, and the tree was constructed by MEGA11.0 using the Neighbor‐Joining method.
**Figure S9 Phylogenetic tree of TGAs from *A. thaliana* and *C. sinensis* .** Protein sequences were aligned by MEGA11.0, and the tree was constructed by MEGA11.0 using the Neighbor‐Joining method.
**Figure S10 Phylogenetic tree of ACTPKs from *Oryza sativa* and *C. sinensis* .** Protein sequences were aligned by MEGA11.0, and the tree was constructed by MEGA11.0 using the Neighbor‐Joining method.
**Figure S11 Interpretation of the transcriptome‐based black‐box model.** (a) Variable importance in the regression model based on the random forest algorithm for each phenotype. Data and error bars represent the mean ± SD (100 repetitions). (b) Partial dependence plots for the regression model based on the random forest algorithm for each phenotype. Blue curves represent LOESS smoothing lines (100 repetitions). Feature values are the z‐scores of gene expression levels.

## Data Availability

All datasets supporting the conclusions of this article are included in the article and supplementary files. Raw data can be accessed from the DDBJ sequence read archive (accession numbers DRR612009–DRR612068). Data have also been deposited in the DDBJ BioProject database (BioProject accession number RPJDB19024).
