# Supplementary material for: Effect of Ammonium:Nitrate Application Ratios on Growth and Nitrogen Metabolism of Tea Plants ( Camellia sinensis L.)
Source: Plant Direct. 2025 Jun 14;9(6):e70084. doi: 10.1002/pld3.70084 (PMC12166195; doi:10.1002/pld3.70084)
Supplement: Supplementary file 3 — Figure S1 Flow chart of the influx kinetics experiment. Figure S2 Effect of inorganic N sources ratio on amino acid concentration in tea plants. (a, b) Free amino acids concentration in tea plants: (a) new leaves, (b) new roots. Data and error bars are the mean ± SD Plots are replicates (n = 6). Different letters indicate significant differences as determined using Tukey’s HSD test (p < 0.05). Figure S3 Hierarchical clustering analysis of new leaves and new roots. Figure S4 Gene expression of N‐metabolism genes in new leaves. Expression level of N‐metabolism genes in new leaves were shown. Data and error bars are the mean ± SD. Plots are replicates (n = 6). Different letters indicate significant differences as determined using Tukey’s HSD test (p < 0.05). N.S., not significant (p ≥ 0.05). Figure S5 Gene expression of N‐metabolism genes in new roots. Expression level of N‐metabolism genes in new roots were shown. Data and error bars are the mean ± SD. Plots are replicates (n = 6). Different letters indicate significant differences as determined using Tukey’s HSD test (p < 0.05). N.S., not significant (p ≥ 0.05). Figure S6 Phylogenetic tree of NIGTs from Arabidopsis thaliana and Camellia sinensis . Protein sequences were aligned by MEGA11.0, and the tree was constructed by MEGA11.0 using the Neighbor‐Joining method. Figure S7 Phylogenetic tree of STOPs from A. thaliana and C. sinensis . Protein sequences were aligned by MEGA11.0, and the tree was constructed by MEGA11.0 using the Neighbor‐Joining method. Figure S8 Phylogenetic tree of TCPs from A. thaliana and C. sinensis . Protein sequences were aligned by MEGA11.0, and the tree was constructed by MEGA11.0 using the Neighbor‐Joining method. Figure S9 Phylogenetic tree of TGAs from A. thaliana and C. sinensis . Protein sequences were aligned by MEGA11.0, and the tree was constructed by MEGA11.0 using the Neighbor‐Joining method. Figure S10 Phylogenetic tree of ACTPKs from Oryza sativa and C. sinensis . Protein sequenc [file PLD3-9-e70084-s001.pptx]

## Slide 1
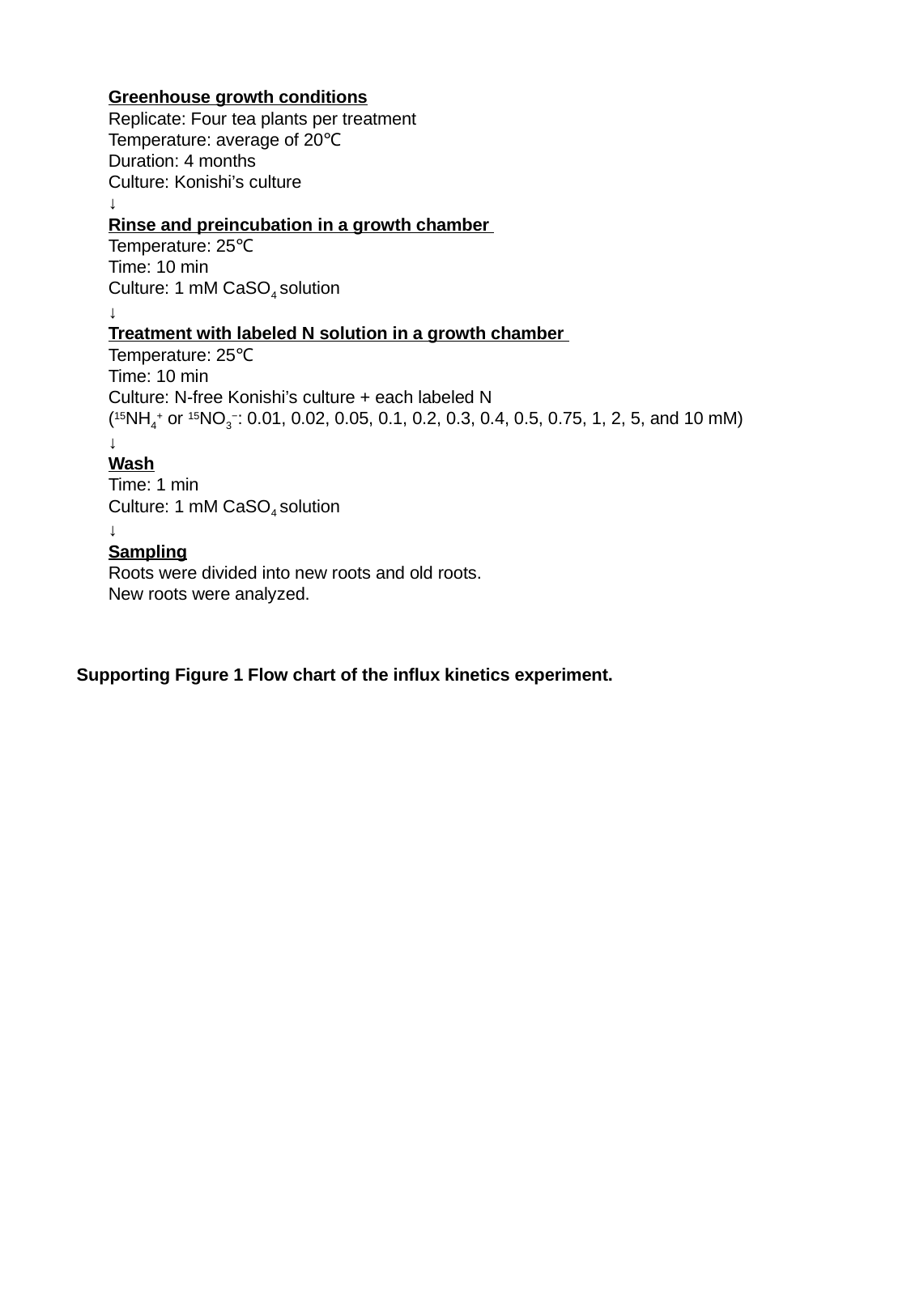

Greenhouse growth conditions
Replicate: Four tea plants per treatment
Temperature: average of 20℃
Duration: 4 months
Culture: Konishi’s culture
↓
Rinse and preincubation in a growth chamber
Temperature: 25℃
Time: 10 min
Culture: 1 mM CaSO4 solution
↓
Treatment with labeled N solution in a growth chamber
Temperature: 25℃
Time: 10 min
Culture: N-free Konishi’s culture + each labeled N
(15NH4+ or 15NO3−: 0.01, 0.02, 0.05, 0.1, 0.2, 0.3, 0.4, 0.5, 0.75, 1, 2, 5, and 10 mM)
↓
Wash
Time: 1 min
Culture: 1 mM CaSO4 solution
↓
Sampling
Roots were divided into new roots and old roots.
New roots were analyzed.
Supporting Figure 1 Flow chart of the influx kinetics experiment.

## Slide 2
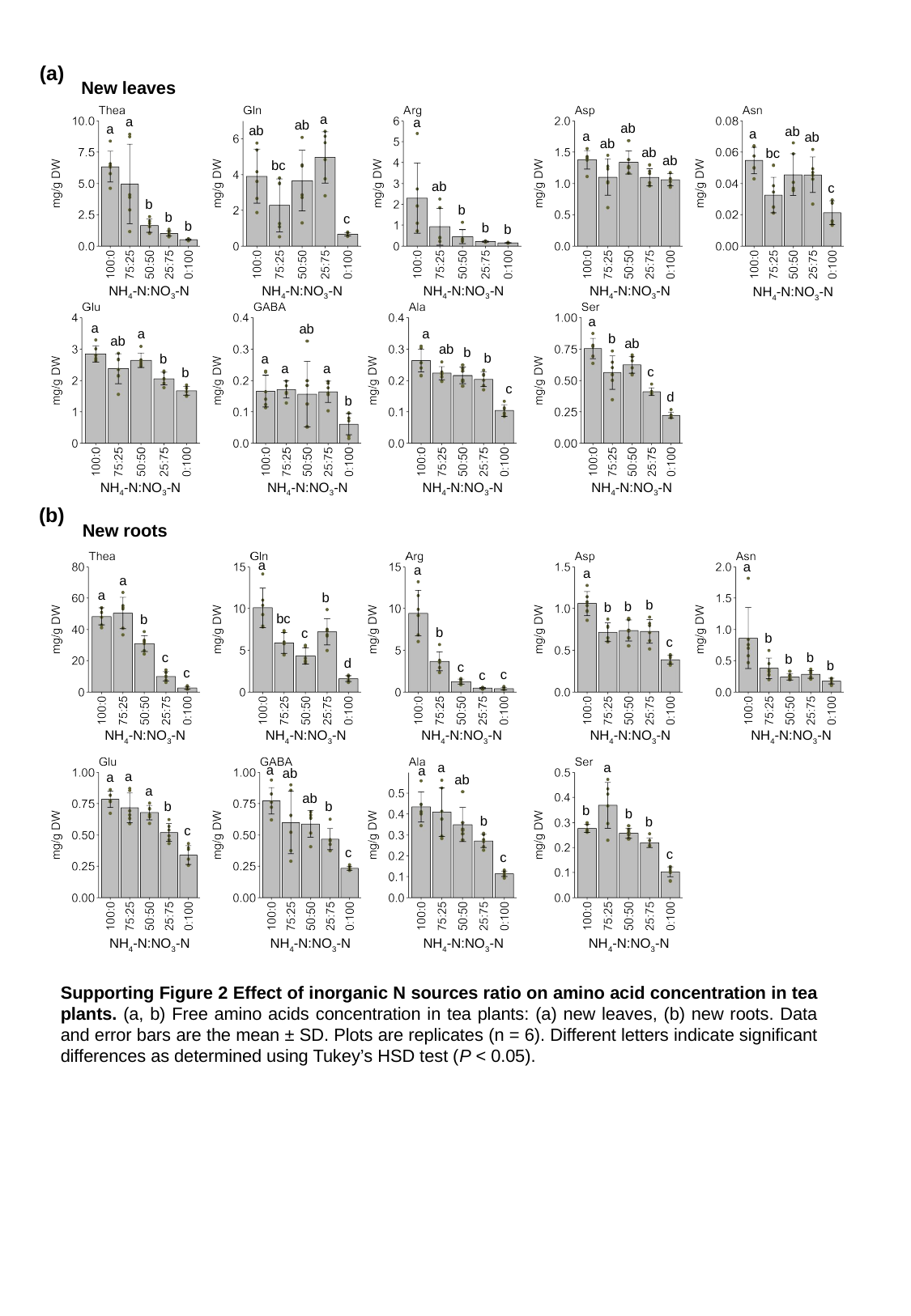

N.S.
(a)
New leaves
a
a
a
ab
ab
a
ab
ab
a
a
ab
ab
ab
bc
ab
bc
ab
c
b
b
b
c
b
b
b
NH4-N:NO3-N
NH4-N:NO3-N
NH4-N:NO3-N
NH4-N:NO3-N
NH4-N:NO3-N
a
a
ab
a
a
b
ab
ab
ab
b
b
a
b
a
a
c
b
c
d
b
NH4-N:NO3-N
NH4-N:NO3-N
NH4-N:NO3-N
NH4-N:NO3-N
(b)
New roots
a
a
a
a
a
a
b
b
b
b
bc
b
b
c
b
c
b
c
b
d
b
c
c
c
c
NH4-N:NO3-N
NH4-N:NO3-N
NH4-N:NO3-N
NH4-N:NO3-N
NH4-N:NO3-N
a
a
a
a
ab
a
a
ab
a
ab
b
b
b
b
b
b
c
c
c
c
NH4-N:NO3-N
NH4-N:NO3-N
NH4-N:NO3-N
NH4-N:NO3-N
Supporting Figure 2 Effect of inorganic N sources ratio on amino acid concentration in tea plants. (a, b) Free amino acids concentration in tea plants: (a) new leaves, (b) new roots. Data and error bars are the mean ± SD. Plots are replicates (n = 6). Different letters indicate significant differences as determined using Tukey’s HSD test (P < 0.05).

## Slide 3
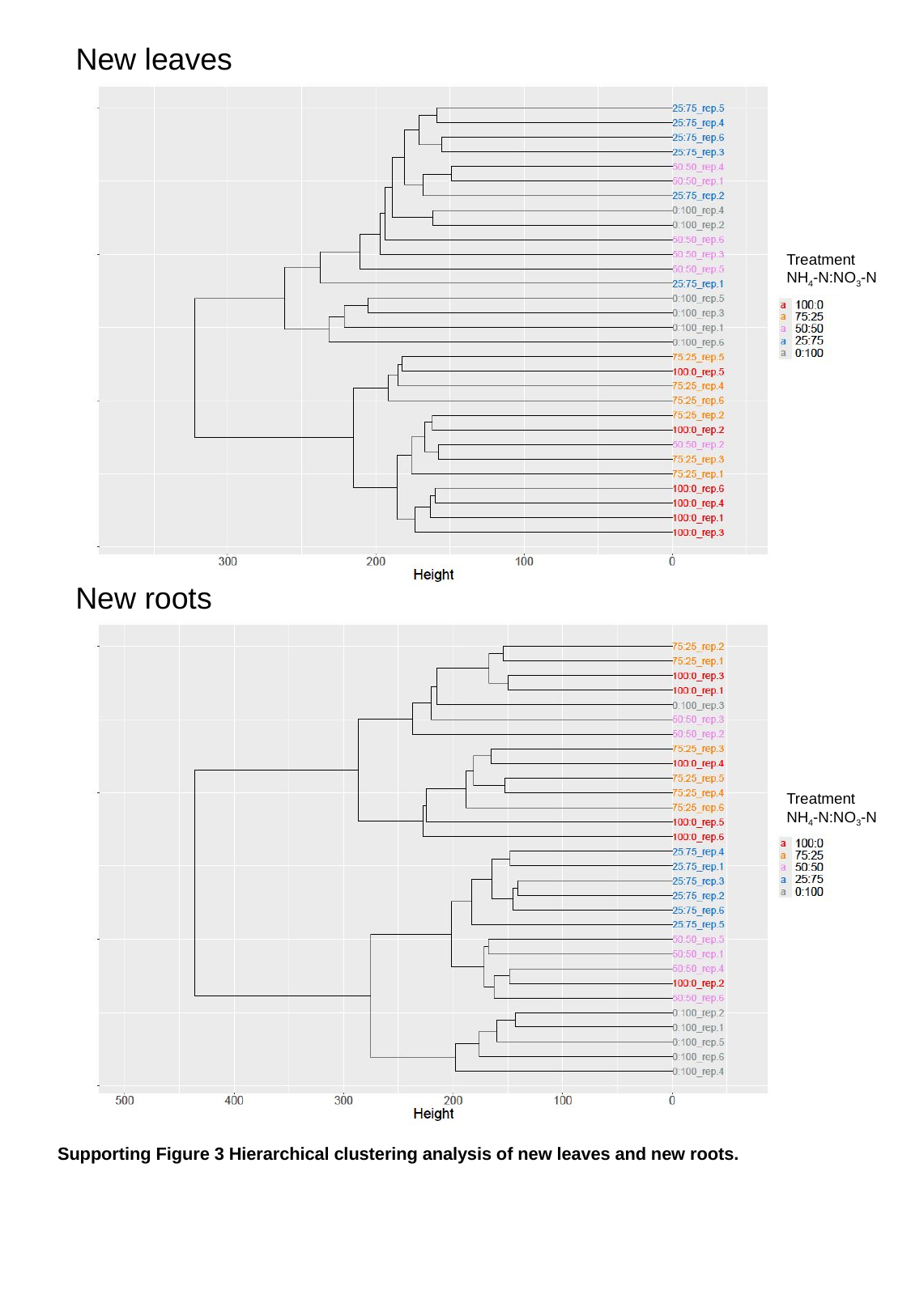

New leaves
Treatment
NH4-N:NO3-N
New roots
Treatment
NH4-N:NO3-N
Supporting Figure 3 Hierarchical clustering analysis of new leaves and new roots.

## Slide 4
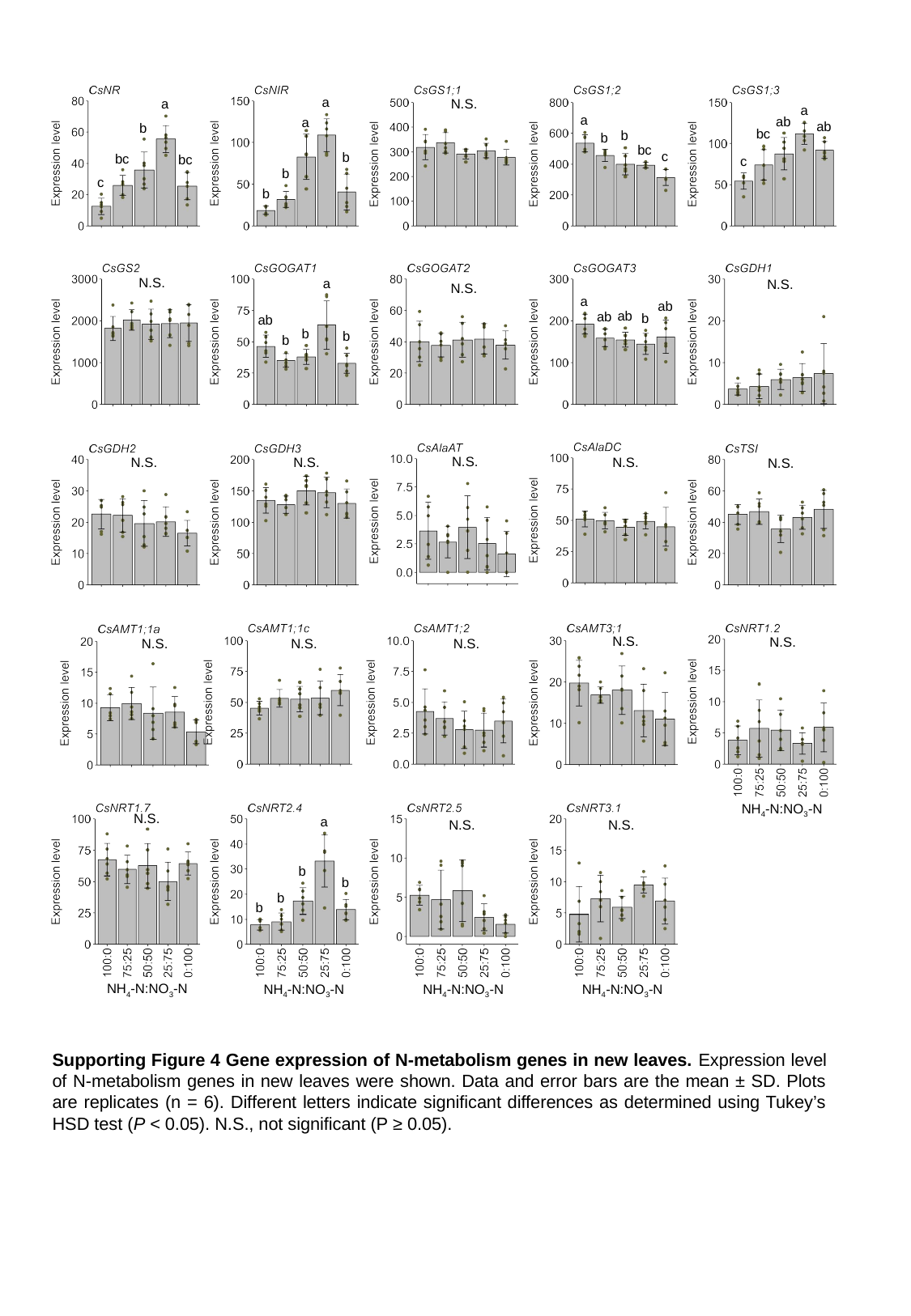

a
a
N.S.
a
a
ab
a
ab
b
bc
b
b
bc
c
b
bc
bc
c
b
c
b
N.S.
a
N.S.
N.S.
a
ab
ab
ab
b
ab
b
b
b
N.S.
N.S.
N.S.
N.S.
N.S.
N.S.
N.S.
N.S.
N.S.
N.S.
NH4-N:NO3-N
N.S.
a
N.S.
N.S.
b
b
b
b
NH4-N:NO3-N
NH4-N:NO3-N
NH4-N:NO3-N
NH4-N:NO3-N
Supporting Figure 4 Gene expression of N-metabolism genes in new leaves. Expression level of N-metabolism genes in new leaves were shown. Data and error bars are the mean ± SD. Plots are replicates (n = 6). Different letters indicate significant differences as determined using Tukey’s HSD test (P < 0.05). N.S., not significant (P ≥ 0.05).

## Slide 5
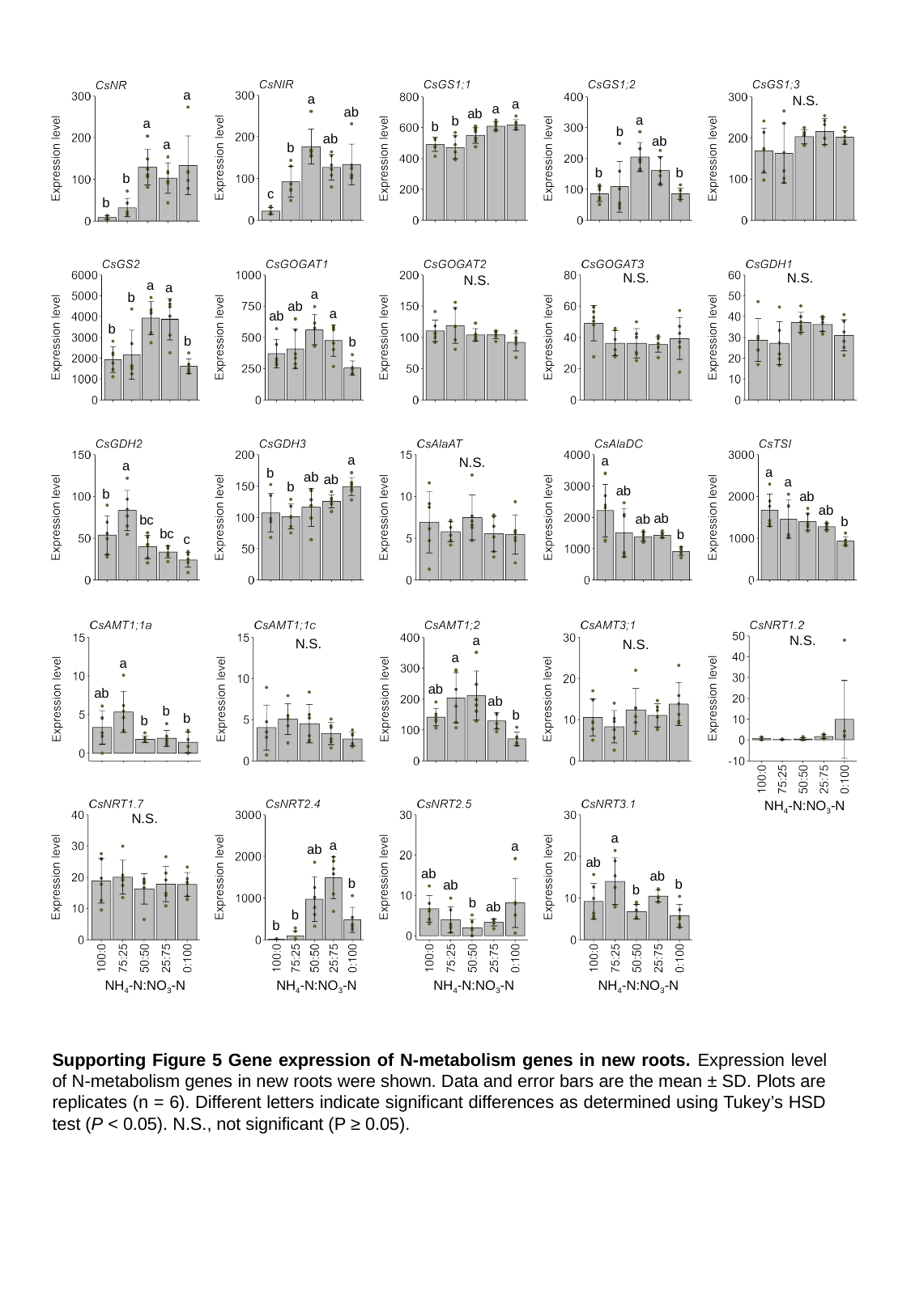

a
a
N.S.
a
a
ab
ab
a
b
a
b
b
ab
ab
a
b
b
b
b
c
b
N.S.
N.S.
N.S.
a
a
a
b
ab
a
ab
b
b
b
a
a
N.S.
a
a
b
ab
ab
a
b
ab
b
ab
ab
ab
ab
bc
b
bc
b
c
a
N.S.
N.S.
N.S.
a
a
ab
ab
ab
b
b
b
b
NH4-N:NO3-N
N.S.
a
a
a
ab
ab
ab
ab
b
b
ab
b
b
ab
b
b
NH4-N:NO3-N
NH4-N:NO3-N
NH4-N:NO3-N
NH4-N:NO3-N
Supporting Figure 5 Gene expression of N-metabolism genes in new roots. Expression level of N-metabolism genes in new roots were shown. Data and error bars are the mean ± SD. Plots are replicates (n = 6). Different letters indicate significant differences as determined using Tukey’s HSD test (P < 0.05). N.S., not significant (P ≥ 0.05).

## Slide 6
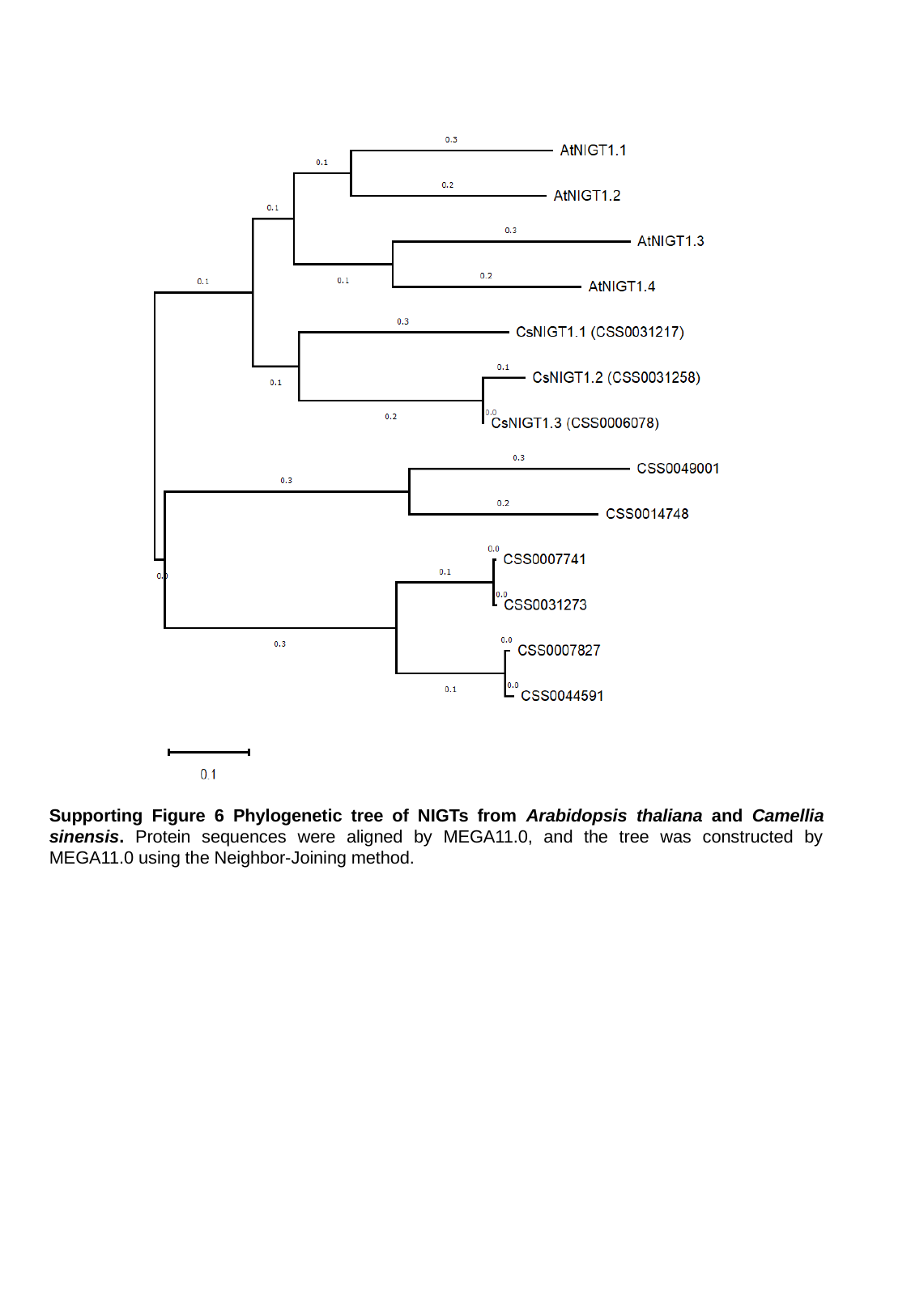

Supporting Figure 6 Phylogenetic tree of NIGTs from Arabidopsis thaliana and Camellia sinensis. Protein sequences were aligned by MEGA11.0, and the tree was constructed by MEGA11.0 using the Neighbor-Joining method.

## Slide 7
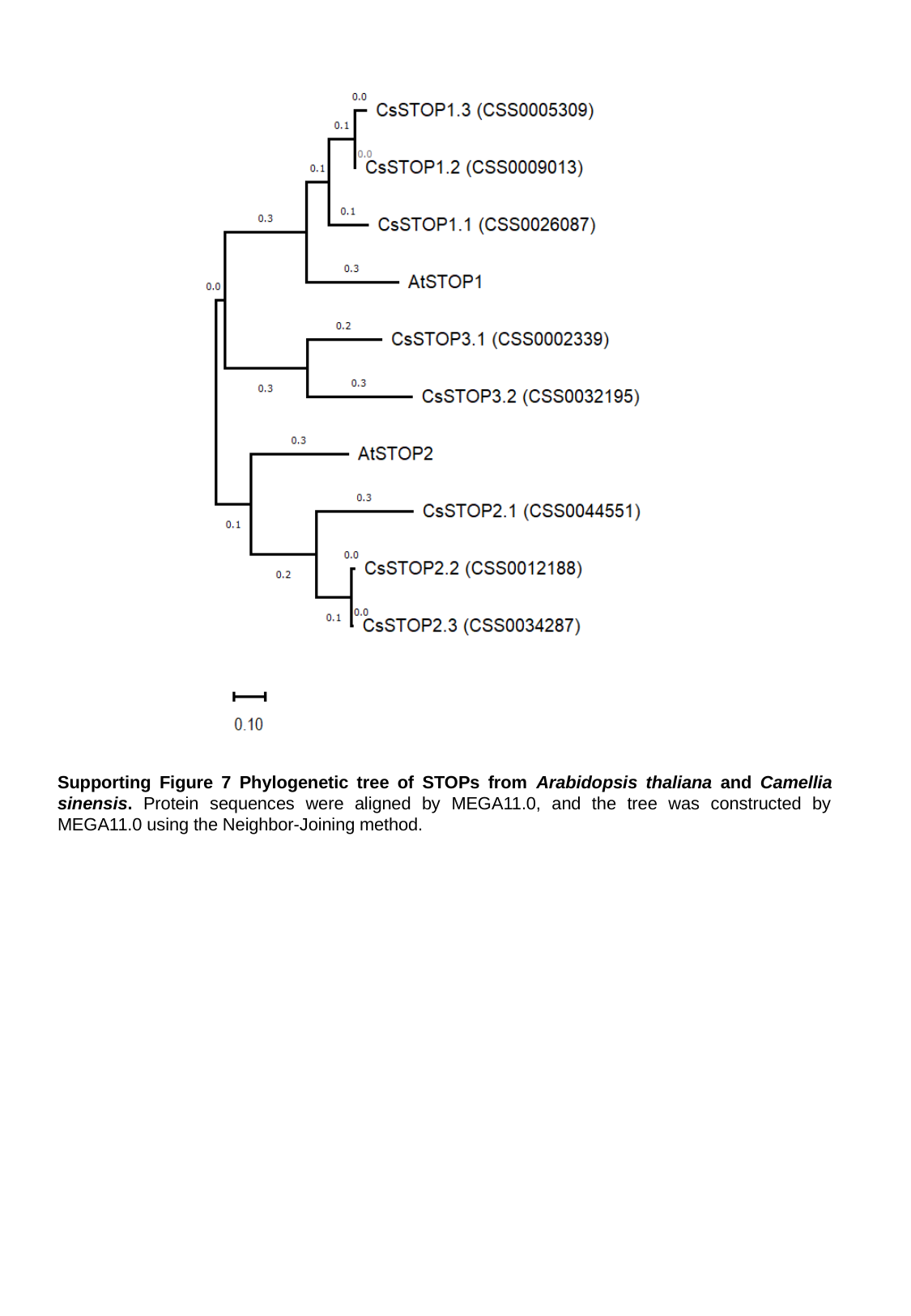

Supporting Figure 7 Phylogenetic tree of STOPs from Arabidopsis thaliana and Camellia sinensis. Protein sequences were aligned by MEGA11.0, and the tree was constructed by MEGA11.0 using the Neighbor-Joining method.

## Slide 8
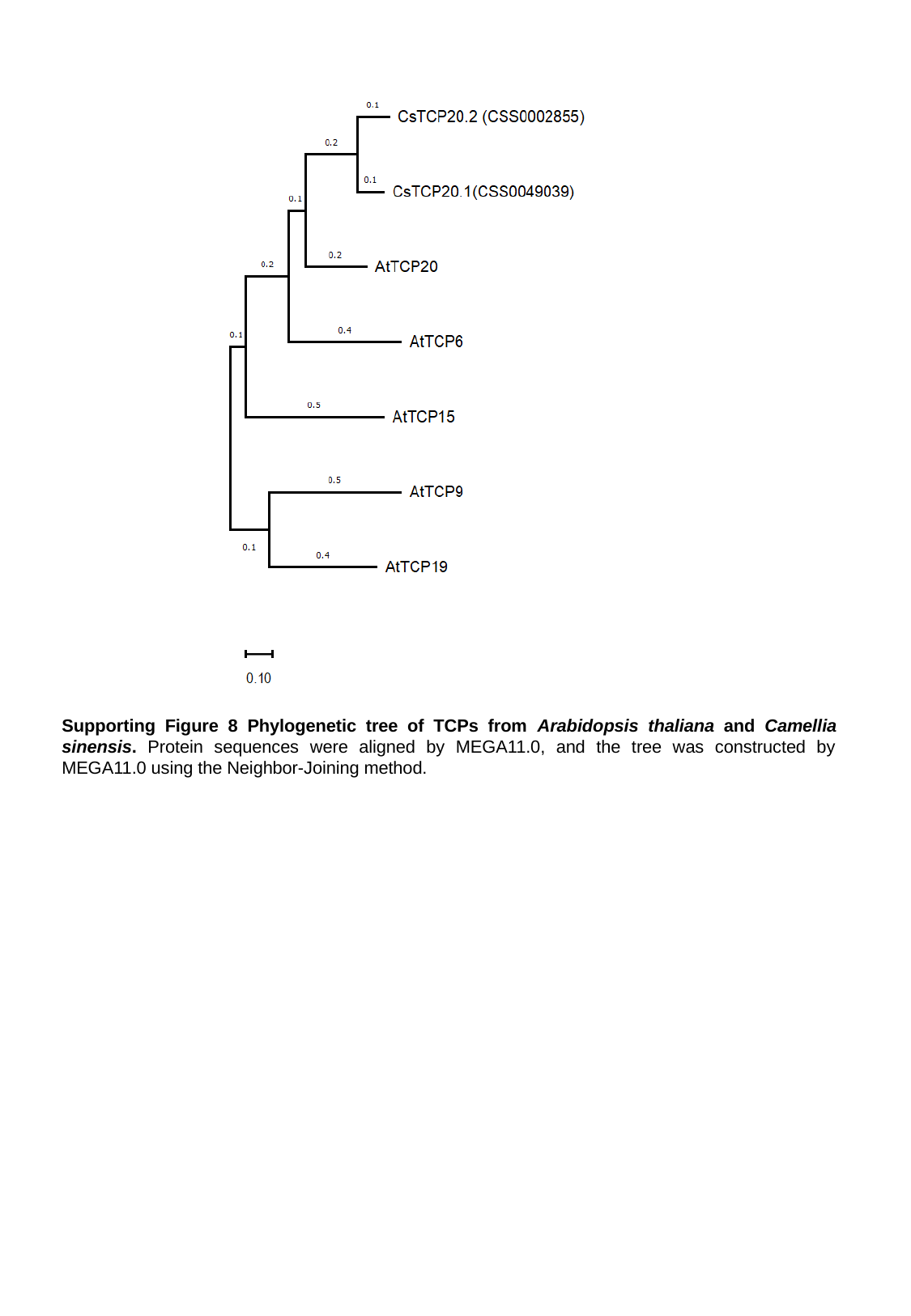

Supporting Figure 8 Phylogenetic tree of TCPs from Arabidopsis thaliana and Camellia sinensis. Protein sequences were aligned by MEGA11.0, and the tree was constructed by MEGA11.0 using the Neighbor-Joining method.

## Slide 9
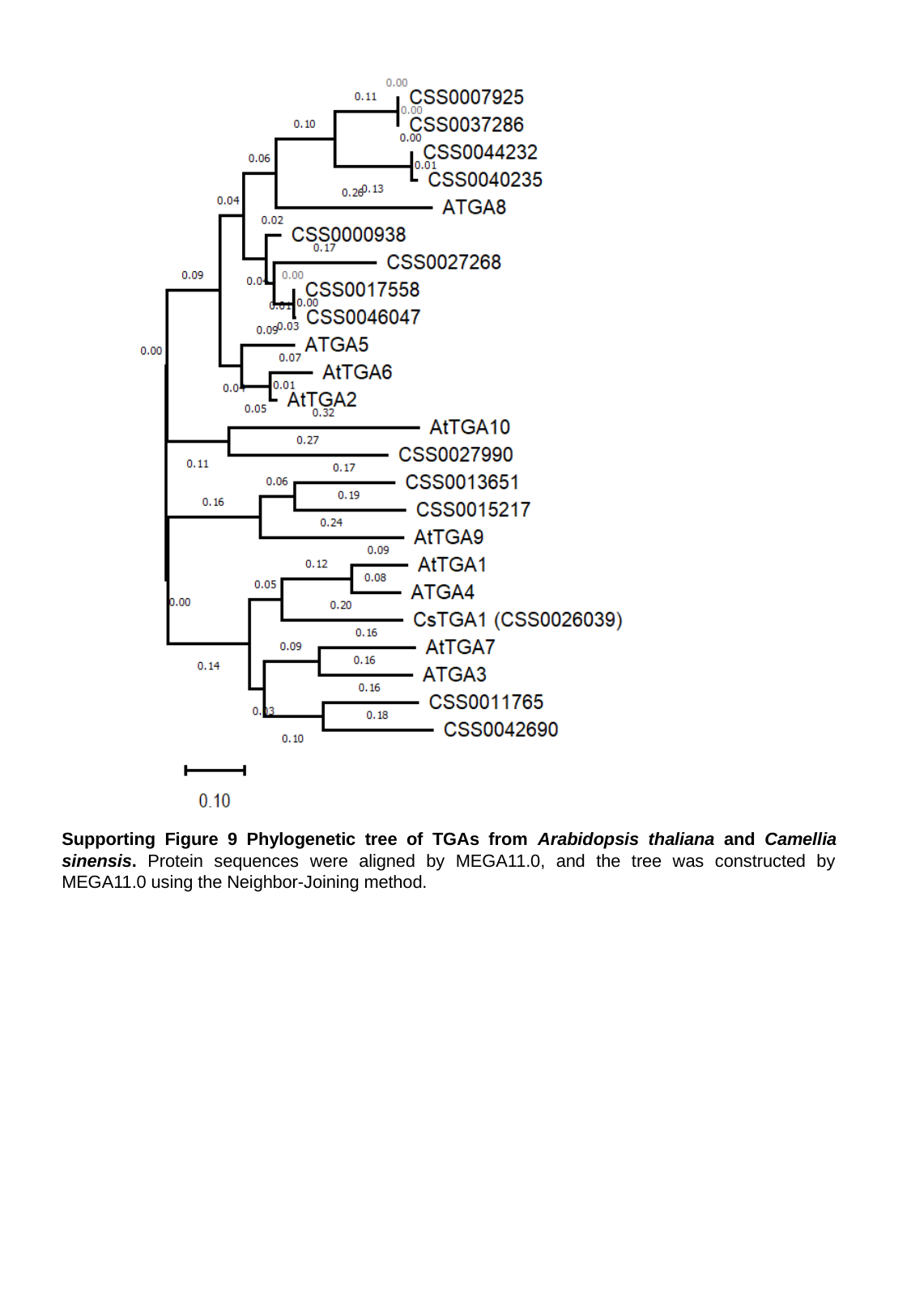

Supporting Figure 9 Phylogenetic tree of TGAs from Arabidopsis thaliana and Camellia sinensis. Protein sequences were aligned by MEGA11.0, and the tree was constructed by MEGA11.0 using the Neighbor-Joining method.

## Slide 10
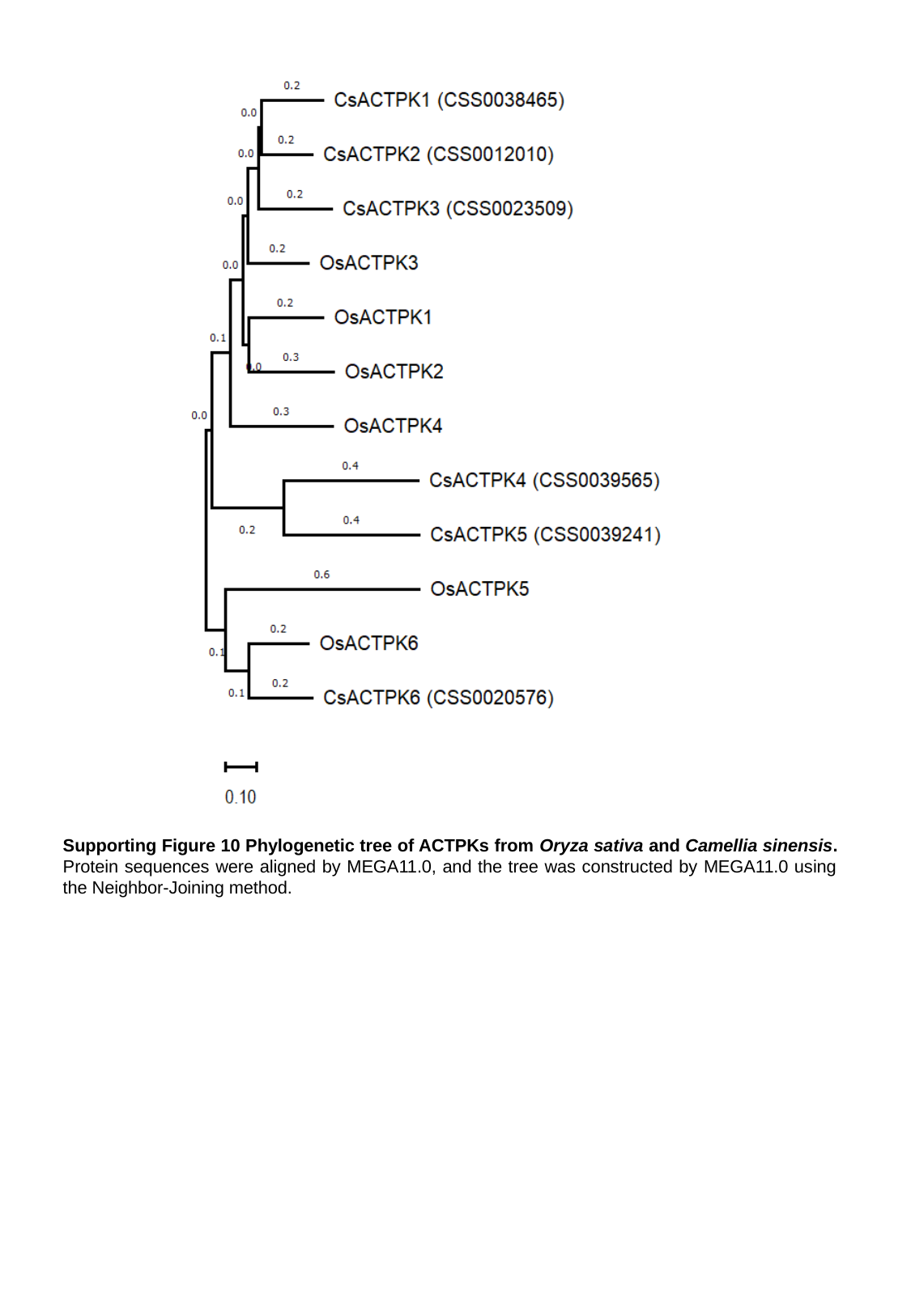

Supporting Figure 10 Phylogenetic tree of ACTPKs from Oryza sativa and Camellia sinensis. Protein sequences were aligned by MEGA11.0, and the tree was constructed by MEGA11.0 using the Neighbor-Joining method.

## Slide 11
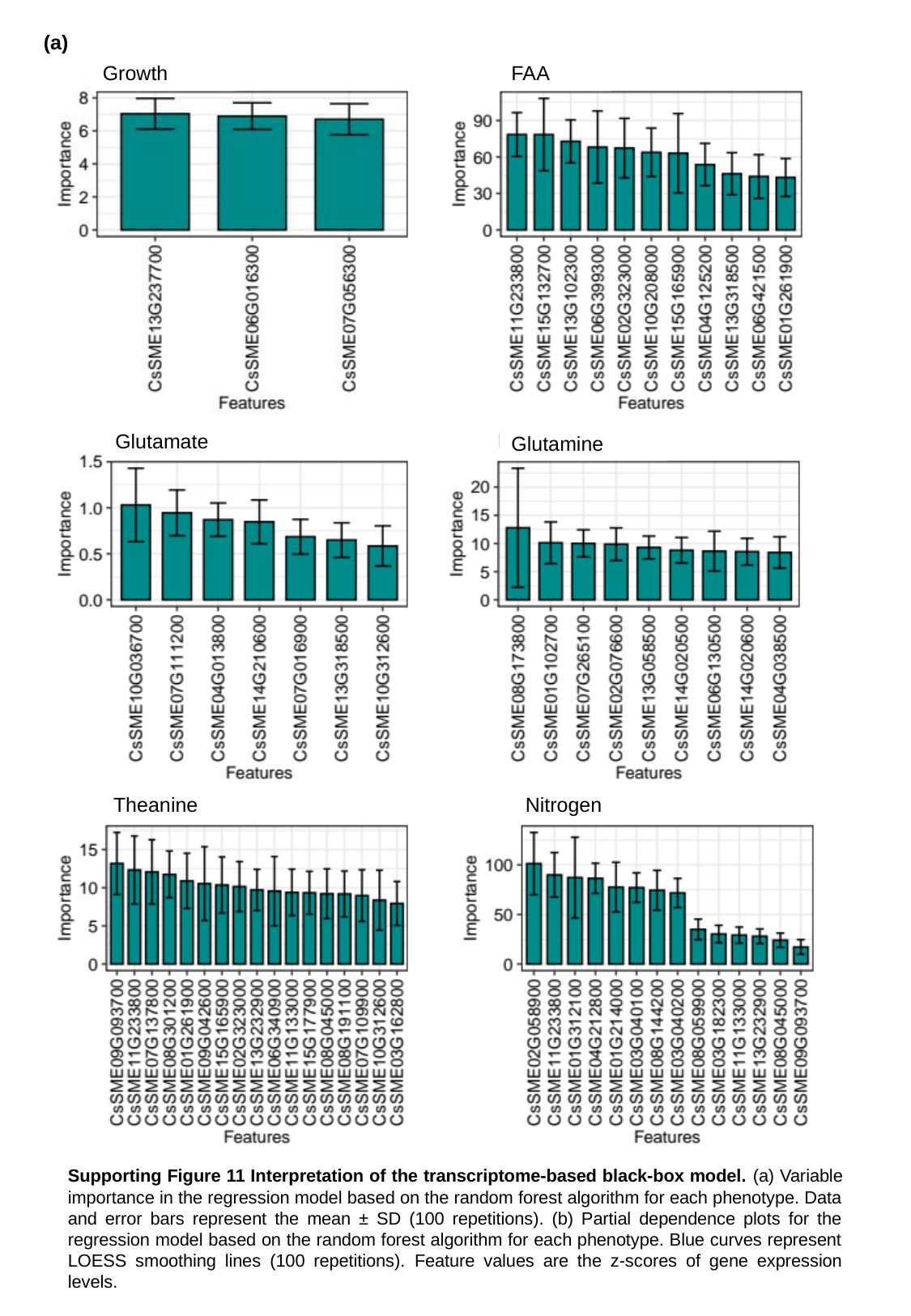

(a)
Growth
FAA
Glutamate
Glutamine
Theanine
Nitrogen
Supporting Figure 11 Interpretation of the transcriptome-based black-box model. (a) Variable importance in the regression model based on the random forest algorithm for each phenotype. Data and error bars represent the mean ± SD (100 repetitions). (b) Partial dependence plots for the regression model based on the random forest algorithm for each phenotype. Blue curves represent LOESS smoothing lines (100 repetitions). Feature values are the z-scores of gene expression levels.

## Slide 12
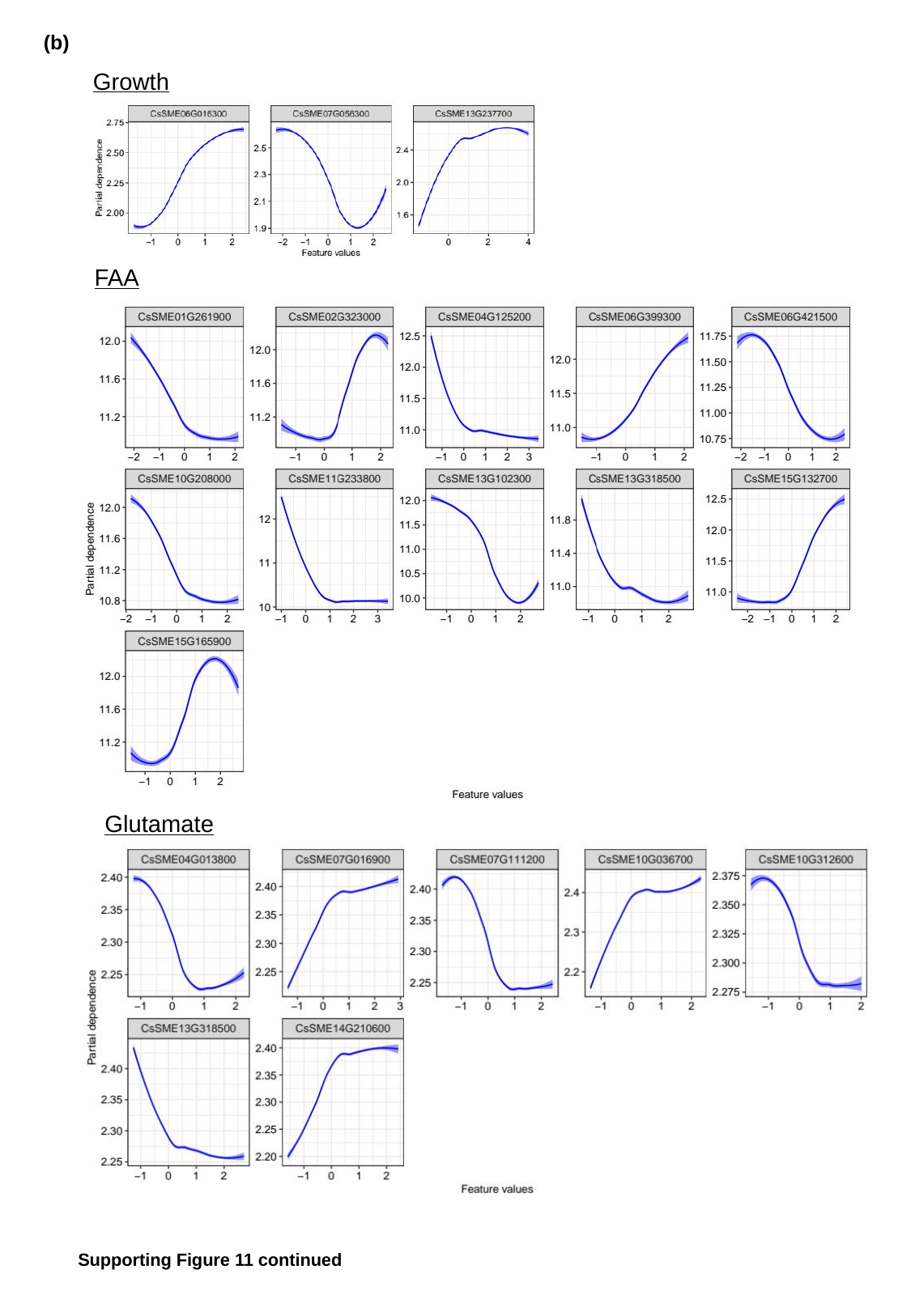

(b)
Growth
FAA
Glutamate
Supporting Figure 11 continued

## Slide 13
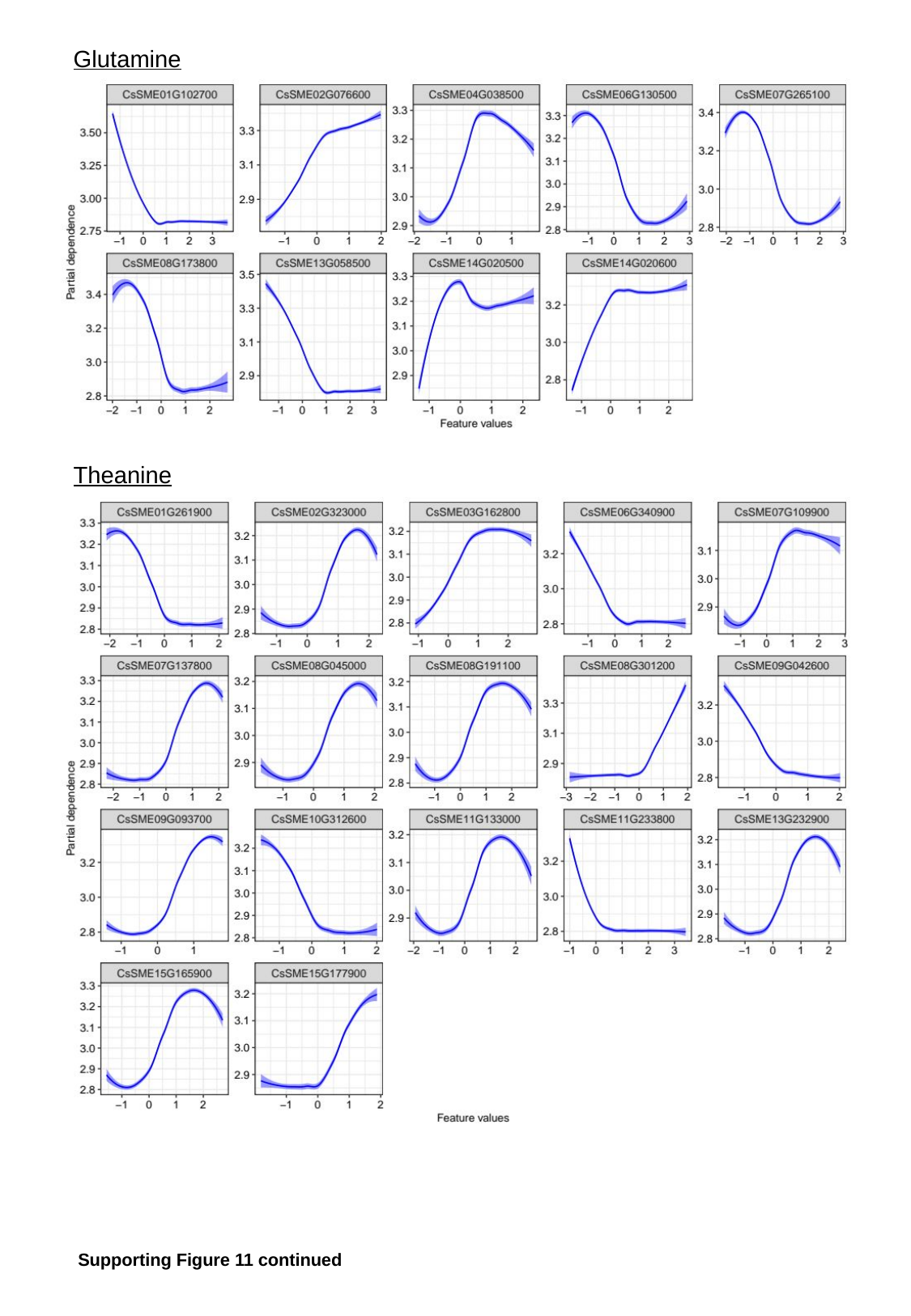

Glutamine
Theanine
Supporting Figure 11 continued

## Slide 14
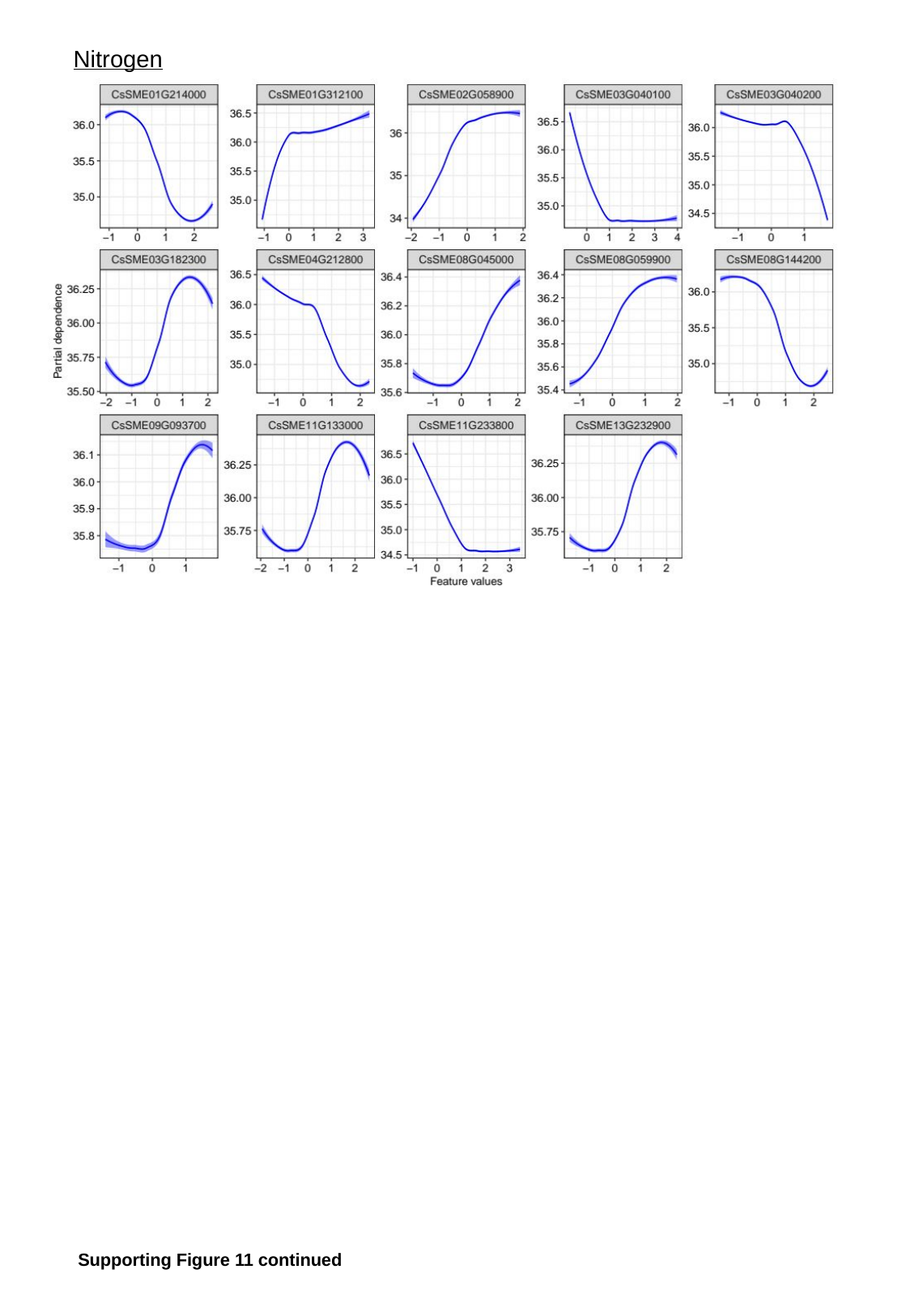

Nitrogen
Supporting Figure 11 continued
