# Supplementary material for: Effect of Ammonium:Nitrate Application Ratios on Growth and Nitrogen Metabolism of Tea Plants ( Camellia sinensis L.)
Source: Plant Direct. 2025 Jun 14;9(6):e70084. doi: 10.1002/pld3.70084 (PMC12166195; doi:10.1002/pld3.70084)
Supplement: Supplementary file 1 — Data S1 Supplementary Peer Review. [file PLD3-9-e70084-s002.pdf]

Plant Direct
Open Access

Effect of ammonium:nitrate application ratios on growth and nitrogen metabolism of tea plants (Camellia sinensis L.)

Dr. Takuo Enomoto, Ms. Natsuki Tone , Mr. Takaya Ishii , Ms. Hisako Hirono , Ms. Ayako Oi , Dr. Yuhei Hirono , Prof. Takashi Ikka , Prof. Hiroto Yamashita

Decision letter round 1

March 27, 2025

Dr. Takuo Enomoto

National Agriculture and Food Research Organization

Institutie of fruit tree and tea science

2769 Kanaya-shishidoi

Shimada, N/A 428-8501

Japan

RE: Effect of ammonium:nitrate application ratios on growth and nitrogen metabolism of tea plants (Camellia sinensis L.)

Dear Dr. Enomoto:

Thank you for submitting to Plant Direct. All required reviews have been returned and we have now finished our evaluation of your manuscript. In light of the reviewers' and editor's comments, further revisions are needed before the paper can be accepted for publication in Plant Direct.

Please view the reviewers' comments below and use their suggestions as a guide while you work on your revision. Note there is an attachment with comments in addition.

When uploading the revised version of this article, please be sure to include the following:

-A word document that contains your response to the reviewers. You should respond to each reviewer comment and note the changes made to the manuscript [the same applies to the comments in the attachment]. If you do not agree with a reviewer's comment and choose not to make a suggested revision, please explain why. Please try to provide as complete an answer as possible to each reviewer's criticisms in the "response to reviewers" section.

-A tracked changes document

- A clean version of the latest version of the manuscript

To upload your revision, please click the link below.

https://plantdirect.msubmit.net/cgi-bin/main.plex?el=A2Lr2ls1A7mhD4I3A9ftdyUP67uEmEvzZ8L1jWuhjOgZ

In order to provide as timely a service as possible, we ask that your revision is resubmitted within three months after receipt of this request. If an extension is needed, please send a request, along with a brief explanation, to the editorial office at plantdirect@wiley.com .

Thank you very much for giving us an opportunity to review your work. I look forward to receiving the next version.

Sincerely,

Jonathan Ingram

Supervising Editor, Plant Direct

-- Reviewer comments:

Reviewer #1:

This study investigated the effects of different nitrate and ammonium combinations as nitrogen sources on the growth, nitrogen composition, and transcriptome of tea plants. The findings highlight the critical role of nitrate and ammonium management in determining tea quality (FAA and theanine levels), yield, and the expression of genes associated with nitrogen metabolism and signaling. Notably, this study identified key candidate compounds and genes that may contribute to tea quality and yield. While these insights enhance practical knowledge for tea cultivation, several aspects require further investigation, as outlined below:

Line 130-132

Please specify the name of the nitrogen compounds used as the nitrate-form and ammonium-form nitrogen sources.

Line 203-210

The indophenol blue method is affected by interference from anthocyanins and amino acids, which can reduce quantification accuracy (Husted et al. 2001 Physiol Plant). Therefore, caution is necessary when applying this method to plant samples. To mitigate this issue, some studies incorporate partial purification steps prior to quantification using the indophenol blue method (Brautigam et al., 2007 Anal Biochem). Improvements are needed for ammonium quantification in the present study.

Line 215-217

Only Figure 1c is described here. Therefore, "(Figure 1b, c)" should be "(Figure 1c)".

Line 274-277

I can see that the the Gln/Glu values for new leaves and new roots are highest at an ammonium:nitrate ratio of 25:75.

Line 289-291

The combined contribution rates of PC1 and PC2 are quite low (Supporting Fig. 2), raising concerns about whether the current PCA analysis adequately captures the overall transcriptomic profile. I recommend that the authors consider alternative methods, such as hierarchical clustering, for sample classification.

Line 362-364, 422-424

It would be more convincing to readers if the authors provided representative gene names associated with "DNA synthesis" and "phosphate ion homeostasis," as gene ontology annotations in the database are often inaccurate.

Line 416-417

If tea plants absorb nitrate but fail to reduce it, nitrate could accumulate excessively within the plants. However, this is not the case (Fig. 3g, h). How do the authors explain this?

Line 453

I think that the following papers are better suited for describing the post-translational regulation of NLPs (Liu et al. 2017 Nature, https://doi.org/10.1038/nature22077; Liu et al. 2022 Science, DOI: 10.1126/science.add1104).

Reviewer #2:

Dear Author,

I have reviewed your manuscript and found it to be an interesting and relevant study. Nitrogen metabolism plays a critical role in tea cultivation, as it significantly influences tea quality. Your investigation into the effects of different ammonium:nitrate ratios on tea growth, free amino acid (FAA) content, and the expression of nitrogen metabolism-related genes provides valuable insights. I have made detailed suggestions and revisions in the manuscript-please review them carefully.

In addition to these specific comments, I have one broader, conceptual suggestion. Given the richness of your phenotypic and RNA-seq data, I recommend performing an integrative analysis using approaches such as LASSO regression or random forest analysis. These methods could help identify key gene-trait associations and strengthen the conclusions of your study.

Decision letter round 2

May 30, 2025

Dr. Takuo Enomoto
National Agriculture and Food Research Organization
Institutie of fruit tree and tea science
2769 Kanaya-shishidoi
Shimada, N/A 428-8501
Japan

Plant Direct
Open Access

MSID: 2024-01674R1
MS TITLE: Effect of ammonium:nitrate application ratios on growth and nitrogen metabolism of tea plants (Camellia sinensis L.)

Dear Dr. Enomoto:

I am pleased to inform you that your manuscript "Effect of ammonium:nitrate application ratios on growth and nitrogen metabolism of tea plants (Camellia sinensis L.)" has been accepted for publication in Plant Direct. Thank you for your careful revision and the commentary provided.

Your article will appear online in the next available issue of Plant Direct. To ensure your article gets published as quickly as possible, please pay attention to the steps detailed below. We have found that most of the delays happen at this stage, especially at the payment stage, so please respond as quickly as possible when prompted.

License Agreement: Once your article has been accepted it will move to Production and undergo admin and file checking - you may receive an email with any queries we have at this stage. When all required items are received by the publisher and queries resolved, the corresponding author will receive an email from Wiley's Author Services system which will ask them to log on at https://authorservices.wiley.com/bauthor and will present them with the appropriate license for completion. Your article cannot be published until both the signed license agreement and payment of the article fee have been received.

Payment of the Open Access Article Publication Fee: All articles published in Plant Direct are fully open access: immediately and freely available to read, download and share. Plant Direct charges a publication fee to cover the publication costs. The corresponding author for this manuscript should have already received a quote from the payments team (cs-openaccess@wiley.com) with the estimated article publication fee; please email cs-openaccess@wiley.com if this has not been received. The corresponding author should log on to the Wiley Author Services site, where the publication fee can be paid by credit card or an invoice. Pro Forma can also be requested. Payment of the publication charge must be received before the article will be published online.

Proofs: You will have the opportunity to look over your paper once more when you receive the author proofs for your article. The proofs will be with you in approximately two weeks. Please note that, in addition to publishing reviewer comments, the author's responses to review comments will also be published alongside the final version of the paper. If you would not like the author's responses to be published, please contact the editorial office at plantdirect@wiley.com .

Promotion of your article: You can help your research get the attention it deserves! Check out Wiley's free Promotion Guide for best-practice recommendations for promoting your work at www.wileyauthors.com/eeo/guide. And learn more about Wiley Editing Services which offers professional video, design, and writing services to create shareable video abstracts, infographics, conference posters, lay summaries, and research news stories for your research at www.wileyauthors.com/eeo/promotion.

Thank you again for your contribution to Plant Direct. If you have any questions, feel free to contact the editorial office at plantdirect@wiley.com .

Best wishes,

Jonathan

Dr Jonathan Ingram

Supervising Editor, Plant Direct

Response to reviewer round 1

28 May 2025 Re:

Manuscript reference No. 2024-01674

We are pleased to submit the revised version of our manuscript, “Effect of ammonium:nitrate application ratios on growth and nitrogen metabolism of tea plants (Camellia sinensis L.),” which we would like to be considered for publication as a Research Article in Plant Direct. We greatly appreciate the constructive comments provided by you and the reviewers. These comments have helped us to significantly improve the quality of our manuscript. In the following pages are our point-by-point responses to each of the reviewer comments. Revisions in the manuscript text are indicated by red font, whereas unchanged text is in black font (i.e., original main text). In the following pages, reviewer comments are provided in blue font. We hope our revisions and point-by-point responses have addressed the concerns that were raised during peer review and that the revised manuscript satisfies the standards for publication in Plant Direct. We look forward to hearing from you at your earliest convenience. Yours sincerely, Takuo Enomoto E-mail: enomotot437@affrc.go.jp, enomoto.takuo320@naro.go.jp Responses to the comments of Reviewer #1 Reviewer comments: Reviewer #1: This study investigated the effects of different nitrate and ammonium combinations as nitrogen sources on the growth, nitrogen composition, and transcriptome of tea plants. The findings highlight the critical role of nitrate and ammonium management in determining tea quality (FAA and theanine levels), yield, and the expression of genes associated with nitrogen metabolism and signaling. Notably, this study identified key candidate compounds and genes that may contribute to tea quality and yield. While these insights enhance practical knowledge for tea cultivation, several aspects require further investigation, as outlined below: 1. Line 130-132 Please specify the name of the nitrogen compounds used as the nitrate-form and ammonium-form nitrogen sources. Response: We appreciate your suggestion. We have added detailed descriptions of the solution compositions in Supplementary Tables 1 to 6. Nitrogen forms are also listed. 2. Line 203-210 The indophenol blue method is affected by interference from anthocyanins and amino acids, which can reduce quantification accuracy (Husted et al. 2001 Physiol Plant). Therefore, caution is necessary when applying this method to plant samples. To mitigate this issue, some studies incorporate partial purification steps prior to quantification using the indophenol blue method (Brautigam et al., 2007 Anal Biochem). Improvements are needed for ammonium quantification in the present study.

Response: Thank you for this valuable suggestion. To prevent anthocyanins and amino acids from interfering with the determination of ammonium concentrations via the indophenol blue method, a pretreatment step involving chloroform and activated charcoal was completed essentially as described by Bräutigam et al. (2007). We revised the Materials and methods section (lines 225– 240) and Results section (lines 299–300) accordingly. 3. Line 215-217 Only Figure 1c is described here. Therefore, "(Figure 1b, c)" should be "(Figure 1c)". Response: We appreciate your suggestion. We revised the text from “(Figure 1b, c)” to “(Figure 1c)” (line 272). 4. Line 274-277 I can see that the the Gln/Glu values for new leaves and new roots are highest at an ammonium:nitrate ratio of 25:75. Response: We appreciate your comment. We apologize for our error. We revised the text from “the Gln/Glu values for new leaves and new roots were highest at an ammonium:nitrate ratio of 75:25” to “the Gln/Glu values for new leaves and new roots were highest at an ammonium:nitrate ratio of 25:75” (line 327). 5. Line 289-291 The combined contribution rates of PC1 and PC2 are quite low (Supporting Fig. 2), raising concerns about whether the current PCA analysis adequately captures the overall transcriptomic profile. I recommend that the authors consider alternative methods, such as hierarchical clustering, for sample classification. Response: Thank you for raising this potential issue with the principal component analysis. To address this issue, we performed a hierarchical clustering analysis to replace the principal component analysis. The results of this new analysis are provided in Supporting Figure 3 of the revised manuscript and are mentioned in the main text (lines 341–345). 6. Line 362-364, 422-424 It would be more convincing to readers if the authors provided representative gene names associated with "DNA synthesis" and "phosphate ion homeostasis," as gene ontology annotations in the database are often inaccurate. Response: We agree with your comment. We revised the main text (lines 424–417 and 506–510) by adding representative gene names and functions. 7. Line 416-417 If tea plants absorb nitrate but fail to reduce it, nitrate could accumulate excessively within the plants. However, this is not the case (Fig. 3g, h). How do the authors explain this? Response: You have asked an important question. Increases in the nitrate proportion will likely be accompanied by increases in nitrate accumulation in new roots. This trend is consistent with the expression level of CsNRT2.4, which substantially contributes to nitrate transport in tea plants. The lack of excessive accumulation in new leaves may be explained by changes in CsNR and CsNIR expression levels. This information has been added to the manuscript (lines 527–535). 8. Line 453 I think that the following papers are better suited for describing the post-translational regulation of NLPs (Liu et al. 2017 Nature, ; Liu et al. 2022 Science, DOI: 10.1126/science.add1104). Response: We appreciate your suggestion. We revised the main text (line 545) to incorporate the suggested references. Responses to the comments of Reviewer #2 Reviewer #2: 1. Dear Author, I have reviewed your manuscript and found it to be an interesting and relevant study. Nitrogen metabolism plays a critical role in tea cultivation, as it significantly influences tea quality. Your investigation into the effects of different ammonium:nitrate ratios on tea growth, free amino acid (FAA) content, and the expression of nitrogen metabolism-related genes provides valuable insights. I have made detailed suggestions and revisions in the manuscript-please review them carefully. In addition to these specific comments, I have one broader, conceptual suggestion. Given the richness of your phenotypic and RNA-seq data, I recommend performing an integrative analysis using approaches such as LASSO regression or random forest analysis. These methods could help identify key gene-trait associations and strengthen the conclusions of your study. Response: Thank you for these insights. In LASSO, variable selection is random, making it difficult to interpret the association with the transcriptome. Furthermore, a causal analysis via random forests does not work well with transcriptomic data

containing many variables. Therefore, we combined a Boruta-based variable selection with a random forest-based causal analysis to examine the association between the phenotype and the transcriptome. We revised the Materials and methods section (lines 245–264), Results section (lines 420–446), and Discussion section (lines 548– 563) accordingly. 2. The glutamine:theanine ratio was higher at an ammonium:nitrate ratio of 25:75 than at 100:0 and 75:25. This suggests that increasing the proportion of ammonium promotes theanine synthesis, whereas increasing the proportion of nitrate enhances glutamine synthesis. You cannot conclude that glutamine and theanine synthesis can be manipulated at will based solely on the observation that the glutamine:theanine ratio was highest at 25:75. This only describes the peak value, not the transition point or the dynamics leading to that ratio. Response: Thank you for providing these insights. To address this comment, we modified this statement in the Abstract (lines 41–42) to reflect the possibility that the ammonium-to-nitrate ratio affects the glutamine synthesis activity-to-theanine synthesis activity ratio. 3. including SENSITIVE TO PROTON RHIZOTOXICITY 3.1 and NITRATE-INDUCIBLE GARP-TYPE TRANSCRIPTIONAL REPRESSOR 1.2, in tea plants. How do these two genes respond to nitrogen availability? Response: You have asked an important question. We added a sentence (lines 45–46) to clarify the roles of these two genes. 4. Theanine synthesis increases with increasing ammonium fertilization because of an increase in THEANINE SYNTHETASE I (CsTS I) expression. Shouldn't there be a supporting reference for this statement? Response: We appreciate your comment. We apologize for confusing theanine concentration with CsTS I expression. We deleted the original sentence and clarified our description of CsTS I as follows: “In tea plants, theanine biosynthesis is mediated by the enzyme encoded by THEANINE SYNTHETASE I (CsTS I), with the CsTS I expression pattern closely correlated with the theanine concentration (She et al., 2020). Theanine concentrations increase with increases in the application of ammonium (Ishigaki, 1978). This indicates that ammonium fertilization may lead to increased CsTS I transcription” (lines 79–84). 5. Materials and methods The methods section lacks statistical analysis. Response: We appreciate your comment. We added a new Statistical analysis section (lines 242–264) that describes how data were processed and analyzed (e.g., regression modeling). 6. A slightly modified version of the culture method described by Konishi et al. (1985) was used. Please provide more details regarding the modified version to enhance clarity and reproducibility. Response: Thank you for this valuable suggestion. We added new Supplementary Tables 1 to 6, which provide details regarding solutions. The solution described by Konishi et al. (1985) was modified in several ways according to changes in nutrient contents (e.g., calcium) that occur when the nitrogen source is changed. 7. cultivar, were transplanted to Wagner pots (size: 1/5,000; diameter: 159 mm; depth: 190 mm) containing 3 L tap water adjusted to pH 4.2 and continuously aerated. Was aluminum included in the nutrient solution? Response: The solution contained aluminum. Details regarding solution compositions are provided in Supplementary Tables 1 to 6. 8. Treatment solutions were renewed weekly. One week or two weeks? Response: We renewed the treatment solutions every week. We replaced “…renewed weekly” with “…renewed once per week” (line 138). 9. new leaves, new stems, mature leaves, mature stems, old roots, and new roots The description would be clearer if the number of leaves is specified from the top, for example: new leaves (1st–3rd leaves from the top) and mature leaves (4th–6th leaves from the top). Response: We appreciate your suggestion. We revised the text to describe the sampling sites more clearly (lines 140–143). 10. two shoots What is the top two shoots? Response: It refers to two new shoots at the top. We replaced “…new leaves from the top two shoots” with “…leaves from two new shoots at the top…” (lines 143–145). 11. Plants used for the kinetics analysis were grown for 4

months as described by Konishi et al. (1985). What are the differences between this method and the modified version? Response: The original method used ammonium nitrate as NO3-N, whereas the modified method used calcium nitrate, potassium nitrate, and magnesium nitrate as NO3-N. Details are provided in Supplementary Tables 1 to 6. 12. Four tea plants Were only four tea plants used in total, or were four plants grown per pot? Response: We used four tea plants per treatment, with each plant grown in a separate pot. We replaced "Four tea plants…" with "Four replicates of tea plants per treatment…" (line 153). 13. They were transferred to nutrient solutions containing either (15NH4)2SO4 (15N abundance = 99.9%) or K15NO3 (15N abundance = 99.9%) as the only N source and incubated in a growth chamber for 10 min. External 15NH4+ or 15NO3− concentrations in this solution were 0.01, 0.02, 0.05, 0.1, 0.2, 0.3, 0.4, 0.5, 0.75, 1, 2, 5, and 10 mM. It is difficult to understand how the experiment was conducted based on the current description. Key information is missing, including the number of replicates, treatment duration, and the rationale behind the treatment. Please provide these details to improve clarity and reproducibility. Response: We appreciate your suggestion. We revised the text (lines 151–161) to address your comment. Additionally, we added a new Supporting Figure 1, which presents a flow chart of the method to further illustrate how the experiment was completed. The concentrations were determined with reference to Yang et al. (2013). 14. We assumed only the high-affinity transporter was active below a certain substrate concentration and the low-affinity transporter was activated above a certain substrate concentration. What evidence supports the assumption that there are two distinct uptake mechanisms in tea plants? Response: Yang et al. (2013) analyzed Chinese tea varieties and presented data suggesting the existence of high-affinity and low-affinity transporters. We revised the text and cited the study by Yang et al. (2013) (lines 171–172). 15. α What does it mean? Response: We appreciate your question. In this case, α refers to the substrate concentration. To avoid any confusion, we changed α to S. We also added the following to the text: "S is the substrate concentration…" In addition, part of the formula and Figure 2e have been modified (lines 179–180). 16. new leaves and new roots? Response: We extracted RNA from new leaves and new roots using separate RNA extraction kits because the extraction efficiency differed between new leaves and new roots (lines 185-189). 17. at different ratios What is the control group? Response: An ammonium:nitrate ratio of 75:25 was used as the control. This ratio is the same as that used by Konishi et al. (1985). We added the following to the manuscript: "…the control ammonium:nitrate ratio (75:25) was set on the basis of the control used by Konishi et al. (1985)" (lines 268–271). 18. The total N concentration in new leaves and new roots tended to increase as the proportion of ammonium increased (Figure 3a, b). The total N content in new leaves and new roots peaked at an ammonium:nitrate ratio of 25:75 (Figure 3c, d). Which nitrogen-related parameters are considered the most reliable in this study? Response: Thank you for asking this question. Both N-related parameters (i.e., concentration and content) are considered important. Specifically, the N concentration is correlated with the amino acid concentration, whereas the N content is correlated with the growth of new leaves and new roots. We added the following to the manuscript: "The N concentration and content were correlated with the FAA content and growth, respectively (Figure 1d, h, 3a–d, 4a, b)" (lines 451–452). 19. but RNA-seq data for these genes were unavailable in this study. Why? Response: We think that these genes were expressed at low levels. Therefore, they were not analyzed. We added the following to the manuscript: "…possibly because these genes were expressed at low levels" (line 394). 20. Variations in gene expression in new leaves under different N

conditions Changes? Response: We appreciate your comment. We replaced “Variations” with “Changes” (line 405)
